# Modulation of basal cell fate during productive and transforming HPV‐16 infection is mediated by progressive E6‐driven depletion of Notch

**DOI:** 10.1002/path.4917

**Published:** 2017-07-24

**Authors:** Christian Kranjec, Christina Holleywood, Diane Libert, Heather Griffin, Radma Mahmood, Erin Isaacson, John Doorbar

**Affiliations:** ^1^ Department of Pathology University of Cambridge, Tennis Court Road Cambridge UK; ^2^ The Francis Crick Institute Mill Hill Laboratory The Ridgeway, Mill Hill London UK

**Keywords:** HPV, E6, Notch, NICD, p53, differentiation

## Abstract

In stratified epithelia such as the epidermis, homeostasis is maintained by the proliferation of cells in the lower epithelial layers and the concomitant loss of differentiated cells from the epithelial surface. These differentiating keratinocytes progressively stratify and form a self‐regenerating multi‐layered barrier that protects the underlying dermis. In such tissue, the continual loss and replacement of differentiated cells also limits the accumulation of oncogenic mutations within the tissue. Inactivating mutations in key driver genes, such as TP53 and NOTCH1, reduce the proportion of differentiating cells allowing for the long‐term persistence of expanding mutant clones in the tissue. Here we show that through the expression of E6, HPV‐16 prevents the early fate commitment of human keratinocytes towards differentiation and confers a strong growth advantage to human keratinocytes. When E6 is expressed either alone or with E7, it promotes keratinocyte proliferation at high cell densities, through the combined inactivation of p53 and Notch1. In organotypic raft culture, the activity of E6 is restricted to the basal layer of the epithelium and is enhanced during the progression from productive to abortive or transforming HPV‐16 infection. Consistent with this, the expression of p53 and cleaved Notch1 becomes progressively more disrupted, and is associated with increased basal cell density and reduced commitment to differentiation. The expression of cleaved Notch1 is similarly disrupted also in HPV‐16‐positive cervical lesions, depending on neoplastic grade. When taken together, these data depict an important role of high‐risk E6 in promoting the persistence of infected keratinocytes in the basal and parabasal layers through the inactivation of gene products that are commonly mutated in non‐HPV‐associated neoplastic squamous epithelia. © 2017 The Authors. *The Journal of Pathology* published by John Wiley & Sons Ltd on behalf of Pathological Society of Great Britain and Ireland.

## Introduction

Papillomaviruses are a large and heterogeneous group of small non‐enveloped DNA viruses that infect a wide range of vertebrates 1, 2. Human papillomaviruses (HPVs) infect cutaneous and mucosal epithelial sites and according to their tumourigenic potential, are commonly referred to as low‐ or high‐risk HPVs. Cervical cancer is the most prevalent human cancer associated with high‐risk HPV infections 3.

Papillomaviruses show an extreme level of adaptation to the regulatory mechanisms governing keratinocyte differentiation, with viral genes being sequentially activated during this process 4. Among the first viral genes to be expressed are E6 and E7, with these proteins also facilitating viral genome amplification by driving cell cycle re‐entry in the differentiating cells of the upper epithelial layers 4, 5. These effects are a consequence of E6 and E7's interaction with a plethora of host‐encoded proteins, of which p53 and members of the pocket protein family are perhaps the most thoroughly characterized 6, 7. The majority of infections are successfully controlled by the host immune system, with clearance or regression to latency occurring in most individuals. In such instances, viral genomes can persist in the epithelial basal layer with very limited viral gene expression. In a small but important number of individuals, however, the failure of the HPV life cycle triggers the progression from productive to a transforming infection, and the possible progression to cancer. In the cervical epithelium, this condition is characterized by the clonal expansion and persistence of dysplastic epithelial cells 8, 9. According to the Darwinian model of neoplastic evolution, mutant cell clones that acquire a competitive advantage are positively selected to persist 10; and in stratified epithelia, clonal expansion is frequently associated with mutations in *TP53* and *NOTCH1* genes 11, 12, 13.

Notch proteins (Notch 1–4 in mammals) are single‐pass type 1 transmembrane proteins and are critical mediators of keratinocyte differentiation 14, 15, 16. Notch is activated upon cell–cell contact by interaction between the Notch receptor and its ligands (delta and jagged in mammals) expressed on the membrane of signalling cells 17, 18. Receptor activation involves the sequential proteolytic processing of Notch proteins, resulting in cytoplasmic release and nuclear translocation of the Notch intracellular domain (NICD) to initiate Notch target gene transcription 17. In keratinocytes, Notch1 plays a crucial role in restraining proliferative phenotypes: its loss of function leads to the aberrant expression of basal cell markers in the differentiating epithelial layers 16, 19, 20 and its inactivation is an important step in the development of squamous neoplasms 13, 21, 22.

In the present study, we examined the contribution of HPV‐16 E6 in the progression from productive to transforming (i.e. abortive) infection through the modulation of p53 and Notch activity in squamous epithelia. Using a combination of 2D monolayer techniques, organotypic raft culture, and the analysis of clinical material, we describe a mechanism by which the restricted expression of HPV‐16 E6 in the basal layer of the epithelium finely tunes differentiation in productive infections, and drives neoplasia when its expression becomes deregulated though the alteration of keratinocyte cell fate.

## Materials and methods

### Clinical samples

The mode of collection, processing, and patient data‐handling of the clinical samples used in this study have been described previously 9, along with a detailed description of the HPV typing methodologies and the diagnosis and grading regimes used by the panel of pathologists 9. All clinical material used in this study was subject to Institutional Review Board approval at the Jagiellonian University Medical College, Krakow, Poland 9.

### Growth assays

NIKS and all NIKS‐derived cells were seeded at a cell density of 1 × 10^5^ cells per well, in F medium (Sigma, Haverhill, UK) without EGF (236EG‐01 M; Bio‐Techne, Abingdon, UK), on 1 × 10^5^ γ‐irradiated J2‐3 T3 in six‐well plates. One day later (day 1), the plating efficiency was estimated and cells were fed with fresh F medium supplemented with 10 ng/ml EGF (Bio‐Techne). To harvest the cells, feeder cells were first dislodged by a short trypsinization step and NIKS keratinocytes were then collected after a second trypsinization and counted using a Z1 Coulter particle counter (Beckman, Takeley, UK). Unless otherwise specified, total cell numbers/ml for each triplicate were assessed on days 1, 3, 4, 5, 7, and 9.

### Fluorescence‐activated cell sorting (FACS) of differentiating NIKS keratinocytes

A modified protocol was used based on previous studies 23. In brief, NIKS cells were first collected by trypsinization and after centrifugation and washing, cells were resuspended to ∼1 × 10^6^ cells/ml in F medium and fixed in 1.5% PFA (J61899; Alfa Aesar, Lancaster, UK) for 10 min at room temperature. Cells were then recovered by centrifugation (3000 rpm) and permeabilized in ice‐cold methanol (1 × 10^6^ cells/500 µl) (Sigma, Haverhill, UK) at 4 °C for 10 min. Cells were subsequently washed in PBS–1% bovine serum albumin (BSA) and passed gently through a 25G needle (Terumo, Leuven, Belgium) for to five times to avoid the formation of cell clumps. Cells were then incubated (1 × 10^6^ cells/100 µl) with a mouse primary antibody to keratin 10 (Krt10) (PA5‐32459; Thermo Scientific, East Grinstead, UK) for 1 h on ice with occasional agitation. The optimal concentration of Krt10 antibody for use in these assays was determined experimentally to be 1 µg/µl. Cells were subsequently washed in PBS–1% BSA and incubated with Alexa Fluor 488 donkey anti‐mouse secondary antibody (A21202; Life Technologies, Paisley, UK) diluted to 1 µg/ml for 30 min at room temperature. After extensive washing (at least three times in PBS), cells were subjected to FACS sorting using either a DxP8 (Cytek, Ely, UK) or a MoFlo MLS cell sorter (DakoCytomation, London, UK).

### Statistical analysis

Quantitative data were expressed as mean ± SD (shown as error bar) from at least three independent experiments. Differences between groups were examined statistically as indicated (*p < 0.05, **p < 0.01, and ***p < 0.001; all *P* values were two‐sided). All statistics were performed using GraphPad Prism7 software. The statistical test used in each case is stated in the main text or in the figure legends.

The Supplementary materials and methods provides details of the plasmids, cell culture, and transfection; the antibodies used; immunofluorescence and immunohistochemistry; reverse transcription–quantitative PCR (RT‐qPCR); and western blot analyses.

## Results

### Progression from low‐ to high‐grade neoplasia reflects a reduced ability of ‘HPV‐16‐infected’ keratinocytes to differentiate and to respond to contact inhibition signals

**Figure 1:**
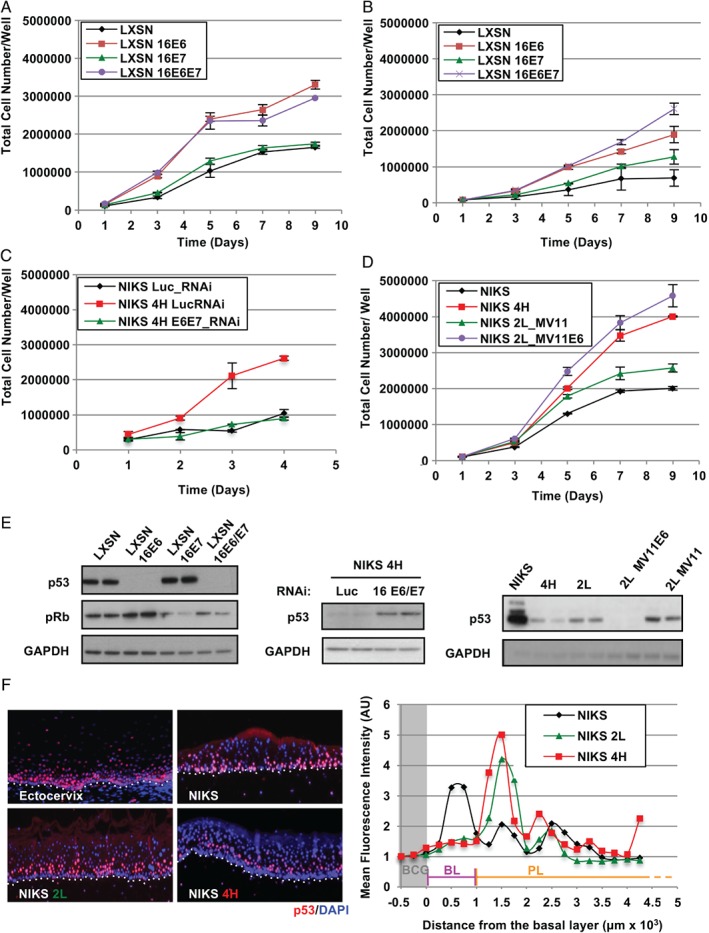
Dominant role of HPV‐16 E6 in driving the growth of NIKS at high cell densities. (A, B) Effects of E6 and E7 expression on the growth pattern of NIKS cells with (A) or without (B) supplemented growth factors. Each point on the plot represents the average from three independent experiments. Error bars represent mean ± SD. (C) NIKS HPV‐16 4H cells were transfected with E6 and E7 RNAi and grown for the indicated time before harvesting and counting. Error bars represent mean ± SD (n = 3). (D) HPV‐16 E6 was ectopically overexpressed in NIKS 2 L (NIKS 2 L MV11_E6) and the effects on cell growth were monitored by growth assay. Error bars represent mean ± SD (n = 3). (E) Representative western blots validating the expression of HPV‐16 E6 and E7 in the experiments in A, C, and D. Levels of p53 and pRb were monitored as surrogate markers for the expression of E6 and E7, respectively. (F) The pattern of expression of p53 was used as a surrogate marker to locate the expression of E6 in raft culture of NIKS 2 L and 4H episomal cell lines. The p53 fluorescence signal was enhanced using tetramethylrhodamine (TMR) tyramide amplification. All sections were counterstained with 4',6‐diamidino‐2‐phenylindole (DAPI). The fluorescent intensity was quantified in the first 0.17 inches of the raft epithelium and normalized with the background signal (BCG) detected immediately underneath the basal layer. BL = basal layer; PL = parabasal layers.

In this study, we used NIKS 24: a near‐diploid, spontaneously immortalized and non‐tumourigenic keratinocyte cell line able to recapitulate the normal keratinocyte differentiation process and the HPV‐16‐mediated 25 life cycle and neoplastic progression in organotypic raft culture 26, 27, 28. To characterize the specific viral functions that may underlie the LSIL (low‐grade squamous intraepithelial lesion) and HSIL (high‐grade squamous intraepithelial lesion) phenotypes observed with full‐length HPV‐16 genomes 27, we generated NIKS cells expressing HPV‐16 E6 and E7, either singularly or in combination (Figure 1). The growth of these cells was subsequently evaluated in monolayer culture over 9 days, during the transition from sub‐confluent (days 1–3) to post‐confluent growth conditions (days 7–9), conditions in which the presence of the full‐length HPV‐16 genome is known to affect normal keratinocyte growth characteristics 27 (Figure 1A). In particular, we were interested to establish whether E6 or E7 was the primary driver of continued cell growth in a confluent basal‐layer‐like environment. When growth factors were present in the medium, 16E7 did not alter the monolayer cell growth rate (Figure 1A; *p* = 0.6245, Student's *t*‐test), with control and E7‐expressing cells reaching confluence around day 5 (∼1 × 10^6^ cells), slowing thereafter as the cell density increased. Under similar conditions, 16E6 conferred a noticeable growth advantage, both when expressed alone (*p* = 0.0149, Student's *t*‐test) and in conjunction with E7 (*p* = 0.0194, Student's *t*‐test; Figure 1A and supplementary material, Figure S1A). Although E7 function appears dispensable in a growth factor‐rich environment, a more obvious role was seen following growth factor depletion (Figure 1B). Here, E7 enhanced the growth rate (*p =* 0.0039, Wilcoxon test) and potentiated the growth‐promoting activity of E6 when the two proteins were expressed together (*p =* 0.0054, Wilcoxon test) (Figure 1B and supplementary material, Figure S1A). Throughout our experiments, E6 stimulated cell growth post‐confluence (Figure 1A, B and supplementary material, Figure S1B), a pattern reminiscent of HSIL‐like NIKS cells harbouring HPV‐16 episomes (NIKS 4H) 27 (supplementary material, Figure S2A, B). Interestingly, when E6 (and E7) levels in the NIKS 4H episomal HPV‐16 cell line were reduced by RNA interference, the cell growth rate fell significantly and approximated that of control NIKS (Figure 1C, E). Conversely, the overexpression of HPV‐16 E6 in the LSIL‐like NIKS line (NIKS 2 L 27) significantly enhanced their growth rate, particularly at high cell density (*p =* 0.03, Student's *t*‐test; Figure 1D, E and supplementary material, Figure S1).

Our results point to a prominent role for 16E6 in overcoming normal keratinocyte contact inhibition in the presence of growth factors, a situation that is found in the epithelial basal layer 7. To investigate this further, organotypic rafts were prepared from the 2 L and 4H episomal cell lines, and E6 activity was assessed using p53 loss as a surrogate marker 6. In NIKS rafts and in normal ectocervical epithelium, p53 expression was prominent in basal and parabasal layers, but declined markedly upon differentiation (Figure 1F). By contrast, NIKS 2 L and 4H were characterized by a progressive increase in basal cell density, in line with their elevated patterns of viral gene expression 27, and a progressively reduced p53 signal in the basal layer of the 4H HSIL raft (Figure 1F). These observations are compatible with the EGF‐mediated regulation of E6 pre‐mRNA splicing 29, which was also apparent in the episomal NIKS lines (supplementary material, Figure S3) and which suggests a specific role for full‐length E6 in modulating basal cell behaviour.

### HPV‐16 E6 abolishes the ability of NIKS to differentiate through Notch inhibition

**Figure 2:**
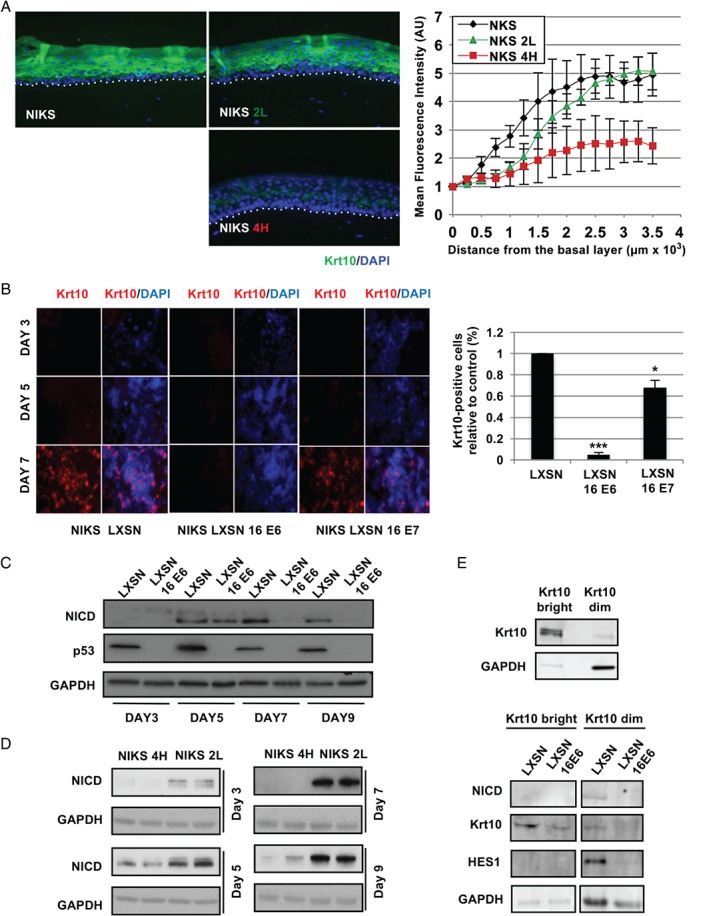
The expression of HPV‐16 E6 prevents the commitment to differentiation of NIKS keratinocytes. (A) Comparison of the timing of expression of the keratin‐10 (Krt10) differentiation marker during epithelial differentiation in organotypic raft cultures of NIKS or the NIKS 2 L (LSIL‐like) and 4H (HSIL‐like) episomal cell lines. Quantification of the Krt10 fluorescence signal during differentiation is shown in the far‐right panel to highlight differences between the different rafts. The fluorescence intensity was normalized against the background signal and plotted against distance from the basal cell layer. Error bars represent mean ± SD (n = 3). (B) Krt10 expression was monitored by immunofluorescence in monolayers of the indicated NIKS cell lines grown to sub‐confluence (day 3), confluence (day 5), and post‐confluence (day 7). The right‐hand panel shows the quantification of Krt10‐positive cells expressed as a percentage of the total cell population at the 7‐day time point relative to the control (LXSN). Ten random fields were acquired for each sample and cells were counted using ImageJ software. (C, D) Western blot analyses of the modulation of NICD by HPV‐16 E6 in NIKS (C) or in NIKS 2 L and 4H (D) across a 9‐day growth assay. (E) Western blot analysis of components of Notch in Krt10‐high and ‐dim FACS‐sorted NIKS cell populations (see also supplementary material, Figure S4).

Inhibition of keratinocyte growth at high cell density occurs as terminal differentiation is triggered 30, 31, 32, 33. In order to examine this in the NIKS cell model, immunostaining was carried out for keratin 10 (Krt10), an established maker of early keratinocyte differentiation 34, 35. In organotypic rafts of the NIKS parental cell line, differentiation occurs in the parabasal cell layers (Figure 2A). In rafts prepared from the NIKS 2 L LSIL‐like rafts, however, Krt10 induction was slightly delayed, with this delay being much more marked in the NIKS 4H HSIL‐like rafts (*p* < 0.0001, one‐way ANOVA). A similar result was obtained with filaggrin, a marker of late squamous differentiation (supplementary material, Figure S2C).

Next, we investigated the implications of 16E6‐mediated abrogation of keratinocyte differentiation in our 2D monolayer model. Keratinocyte differentiation occurred at high cell densities (day 7) in control cells (LXSN‐NIKS) (Figure 2B). Consistent with previous studies 36, the expression of HPV‐16 E7 reduced the levels of Krt10 induction (*p =* 0.0155; Student's *t*‐test), however, and more significantly (*p =* 0.0002, Student's *t*‐test), the expression of HPV‐16 E6 abolished almost completely the induction of Krt10 in post‐confluent NIKS cells (Figure 2B). These results suggest a possible role for E6 in restricting or modulating the rate at which infected cells may be lost from the confluent epithelial basal layer.

Among pathways regulating keratinocyte differentiation, Notch signalling is known to be a strong positive modulator 14, 15, 16. The western blot analysis of Notch cleavage indicated that NICD starts to accumulate at confluence (Figure 2C), consistent with its cell contact‐dependent activation 31. While NICD accumulation was greater and was maintained in post‐confluent (days 7–9) control cells, the expression of HPV‐16 E6 led to its dramatic down‐regulation post‐confluence. Similar results were also obtained when NICD levels were compared in LSIL‐ and HSIL‐like NIKS (Figure 2D). To examine Notch signalling during differentiation, a fluorescence‐activated cell sorting (FACS) approach was used to sort cells based on their Krt10 expression (Krt10‐bright and Krt10‐dim), followed by further analysis by western blotting (Figure 2E, upper panel and supplementary material, Figure S4). In control (LXSN) NIKS, Notch signalling was active in the Krt10‐dim population (Figure 2E, lower panel), whereas the expression of 16E6 markedly reduced the induction of NICD and HES1 expression in both sorted populations, along with the levels of Krt10. This suggests that the activation of Notch signalling occurs transiently in keratinocytes at early stages during the commitment to differentiation. Our data also indicate that loss of NICD is an event associated with increased levels of HPV‐16 E6 expression, supporting a role for E6 in preventing the commitment of keratinocytes to differentiate upon viral life cycle deregulation.

### HPV‐16 E6 requires interaction with p53 but not PDZ proteins to regulate levels of Notch expression

**Figure 3:**
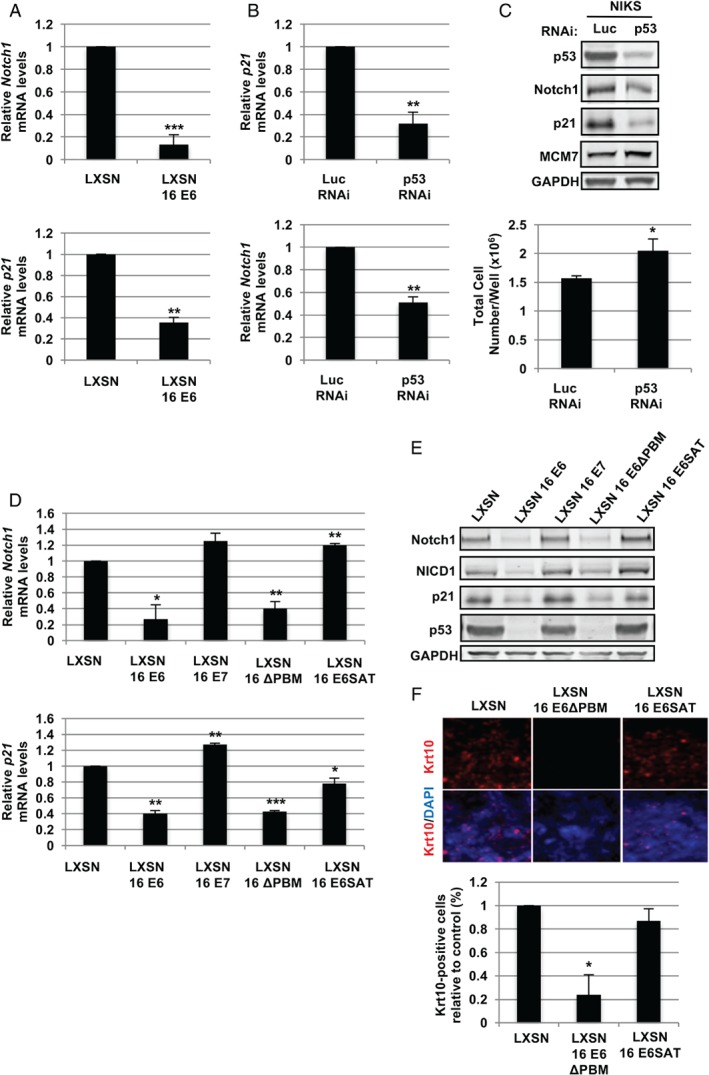
HPV‐16 E6 affects the levels of full‐length and cleaved Notch1 through the degradation of p53. (A, B) RT‐qPCR analysis of the expression of Notch1 and p21 mRNA in control and E6‐expressing cells at post‐confluence (day 7) (A) or in NIKS cells transfected with RNAi to luciferase (control) or p53 (B). Each bar chart represents the average values from three independent experiments. Error bars represent mean ± SD. (C) NIKS cells transfected with RNAi as in panel B were subjected to western blot analysis for the indicated proteins (upper panel). The total cell number of NIKS transfected with control or p53 RNAi was estimated 72 h post‐transfection (see also supplementary material, Figure S5). Error bars represent mean ± SD (n = 3). RT‐qPCR analysis of NOTCH1 and P21 mRNA expression in control NIKS (LXSN) and NIKS expressing E6, E7 or the indicated E6 mutants at post‐confluence (day 7). Error bars represent mean ± SD (n = 3). (E) NIKS cell lines as in panel D were grown to post‐confluence and subjected to western blot analysis for the indicated proteins. (F) Control NIKS or NIKS cells expressing either the wt HPV‐16 E6 or the 16E6 SAT and ΔPBM mutants were grown to post‐confluence prior to fixation. The pattern of Krt10 expression was then analysed by immunofluorescence using Alexa Fluor 594‐conjugated secondary antibodies. The lower panel shows the quantification of Krt10‐positive cells expressed as a percentage of the total cell population at the 7‐day time point relative to the control (LXSN). The quantitative analysis was carried out as in Figure 2B using ImageJ software. Where shown, statistical significance was evaluated using the Student's t‐test.

Previous studies have suggested that E6‐mediated inactivation of Notch may involve p53 37. In order to examine this in NIKS keratinocytes, *NOTCH1* mRNA expression was examined by RT‐qPCR in post‐confluent cells. Interestingly, the expression of E6 led to a more than five‐fold decrease in *NOTCH1* mRNA (Figure 3A), with similar results being obtained for the p53 transcriptional target *P21*. Similarly, the transient ablation of p53 by RNA interference led to a decrease of *P21* mRNA and to an approximately 50% reduction in *NOTCH1* transcripts and protein levels (Figure 3B, C). The ablation of p53 resulted in a marginal increase in mini‐chromosome maintenance‐7 (MCM7) levels (Figure 3C), which might be expected given the increased NIKS cell growth seen following p53 ablation (Figure 3C, lower panel and supplementary material, Figure S5). Previous studies suggested that expression of the p53 homologue p63 maintains the proliferative capacity of keratinocytes 38, 39, 40. Consistent with this, the ablation of p63 in NIKS keratinocytes led to a dramatic reduction of their growth rate (supplementary material, Figure S5).

To further characterize the contribution of HPV‐16 E6 to the regulation of Notch, we repeated *Notch* mRNA and protein analysis in NIKS expressing either wild‐type HPV‐16 E6, a 16E6 mutant lacking the C‐terminal PDZ‐binding motif (ΔPBM), or the 16E6 R8S/P9A/R10T (SAT) mutant, which are unable to bind and degrade PDZ domain‐containing proteins and p53, respectively. Wild‐type HPV‐16 E6 strongly repressed both Notch and p21 at mRNA and protein levels (Figure 3D, E), and was associated with low levels of p53 and NICD (Figure 3E). In the absence of the PDZ binding motif, E6 retained the ability to degrade p53 and significantly inhibited the expression of Notch1 and p21, an ability that was lost in the E6 SAT mutant. Consistently, also E7 failed to down‐regulate Notch expression. The analysis of Krt10 induction in monolayer NIKS expressing E6 mutants confirmed that the ability of E6 to degrade p53 is indeed necessary to prevent commitment to differentiation, whereas the PDZ‐binding defective E6 mutant retains the ability to significantly modulate keratinocyte differentiation (*p =* 0.0162, Student's *t*‐test) (Figure 3F). Taken together, these results support previous observations 37, 41 and allow us to conclude that p53 and Notch1 are crucial regulators of keratinocyte cell fate 12, 13, 21.

### Progression to high‐grade neoplasia correlates with a progressive loss of p53 and NICD1 in HPV‐16 raft cultures

**Figure 4:**
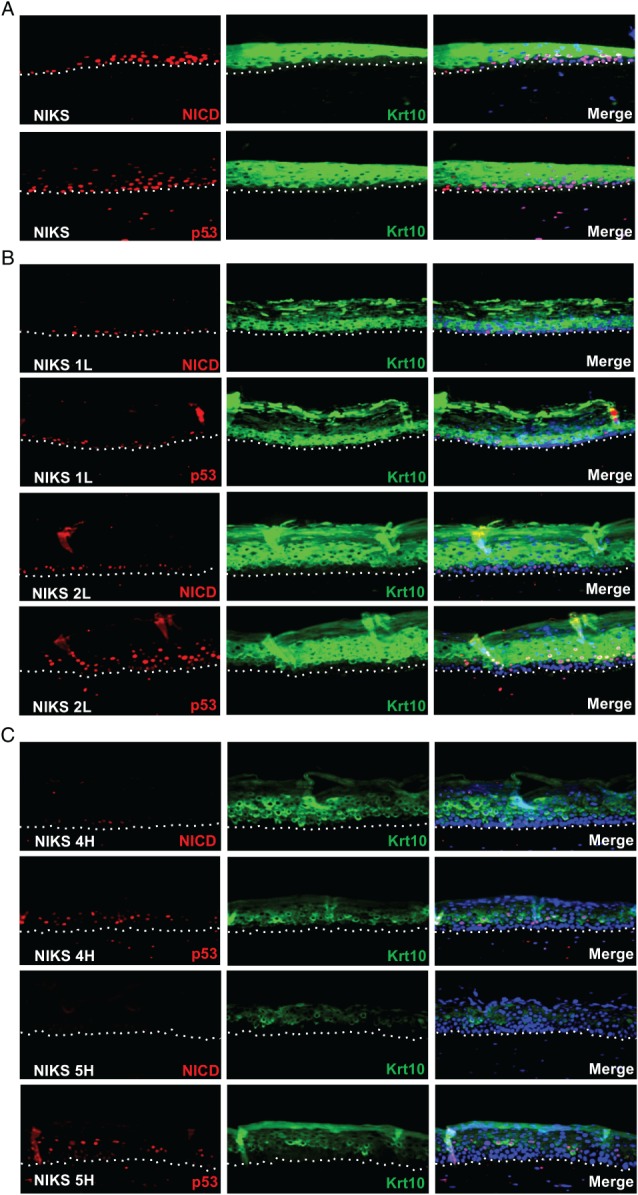
Loss of p53, NICD, and Krt10 expression correlates with abortive infection phenotypes in raft culture. (A–C) Representative images showing the pattern of p53, NICD, and Krt10 expression in organotypic raft culture sections of parental NIKS (A) and LSIL‐like (B) and HSIL‐like (C) NIKS HPV‐16 episomal lines. The fluorescence signal for p53 and NICD was amplified with TMR. Krt10 fluorescence was visualized using Alexa Fluor 488‐conjugated secondary antibodies (see also supplementary material, Figure S9). All sections were counterstained with DAPI.

The restricted 16E6 activity in the basal layer of the raft cultures suggests that p53 and Notch1 are strictly regulated by the virus. To examine their modulation during progression from LSIL to HSIL, the expression of p53, NICD, and Krt10 in organotypic raft cultures was examined (Figure 4; see supplementary material, Figure S6A for H&E images). In parental NIKS rafts, cleaved Notch was occasionally detected in basal cells as well as in the parabasal layers (Figure 4A and supplementary material, Figure S7). This supports an activation of Notch1 at early differentiation stages (Figure 2E) and is in agreement with studies in mice indicating that the fate commitment of basal keratinocytes to differentiate is an early event 42. The pattern of p53 expression was similar to that of NICD (Figure 4A), whereas Krt10 was restricted to differentiating cells of the suprabasal layers. The majority of Krt10‐positive cells were negative for NICD, which agrees with the data presented above (Figure 2E).

In raft culture, LSIL‐like NIKS HPV‐16 clones showed abundant 16E4 and L1 expression (supplementary material, Figure S6B), identifying them as productive phenotypes 27. Among these, NIKS 1 L retained scattered NICD‐ and p53‐positive basal cells (Figure 4B and supplementary material, Figure S7), consistent with the fact that these cells express E6 and E7 at the lowest levels 27. In contrast, NIKS 2 L rafts had a more apparent parabasal expression of p53 and NICD, in line with the higher levels of HPV‐16 E7 seen in these cells 27. In both LSIL‐like raft cultures, Krt10 expression was only marginally affected, with a more evident delay in expression seen in the 2 L rafts (Figure 4B and supplementary material, Figures S7 and S8). These data suggest that in the context of the productive HPV life cycle, the lower E6 activity in the epithelial basal layer allows p53 and Notch to persist and mediate adequate levels of keratinocyte differentiation to support the productive viral life cycle.

An elevation of E6 and E7 expression is suspected during progression from LSIL to HSIL 43, 44. NIKS HPV‐16 HSIL‐like 4H and 5H rafts (Figure 4C) showed a dramatic reduction in both NICD and p53. These proteins were almost undetectable in the basal layer of NIKS 4H rafts, with NICD present only in sporadic parabasal cells. In accordance with this, the appearance of Krt10 was delayed, and its abundance was reduced compared with LSIL‐like and normal NIKS raft cultures. In the higher‐grade rafts (NIKS 5H), NICD was almost undetectable, with a further delay in p53 and Krt10 expression (Figure 4C and supplementary\textbf{a} material, Figures S7 and S8). Consistent with previous studies 9, 27, these observations suggest the progressive expansion of a proliferative basal‐like cell population into the suprabasal epithelial layers. To further corroborate this in our raft culture system, we extended our analysis to include MCM7 and p63, as markers of cell cycle progression 45, and the basal cell compartment 46, 47, respectively (supplementary material, Figure S9A). Rafts prepared from parental NIKS showed a pattern of MCM7 and p63 expression similar to that found in normal stratified epithelia 40, 46, 47, 48. In contrast, in the LSIL‐like rafts and even more dramatically in HSIL‐like rafts, the expression of MCM7 and p63 extended into the parabasal layers of the epithelium (supplementary material, Figure S9A). Notably, the suprabasal expression of ΔNp63, the N‐terminally truncated p63 isoform responsible for the inhibition of p53 activity 49, 50 and keratinocyte differentiation 51, could be observed exclusively in the HSIL‐like 5H NIKS raft cultures (supplementary material, Figure S9A).

To assess more precisely the role of E6 and E7 in modulating basal cell fate, raft analysis was repeated following the expression of these proteins in isolation (supplementary material, Figure S9B, C). Cleaved Notch1 and Krt10 were drastically depleted in E6‐expressing rafts, whereas E7 expression reduced Krt10 accumulation slightly, but did not obviously perturb NICD (supplementary material, Figure S9B). Conversely, E6 expression led to an enhancement of MCM7 limited to the basal and parabasal layers when compared with control rafts (supplementary material, Figure S9B), suggesting that, in contrast to E7, E6‐driven modulation of cell fate occurs in the basal compartment.

### Disrupted Notch1 activation is a characteristic of HPV‐16‐positive cervical lesions

**Figure 5:**
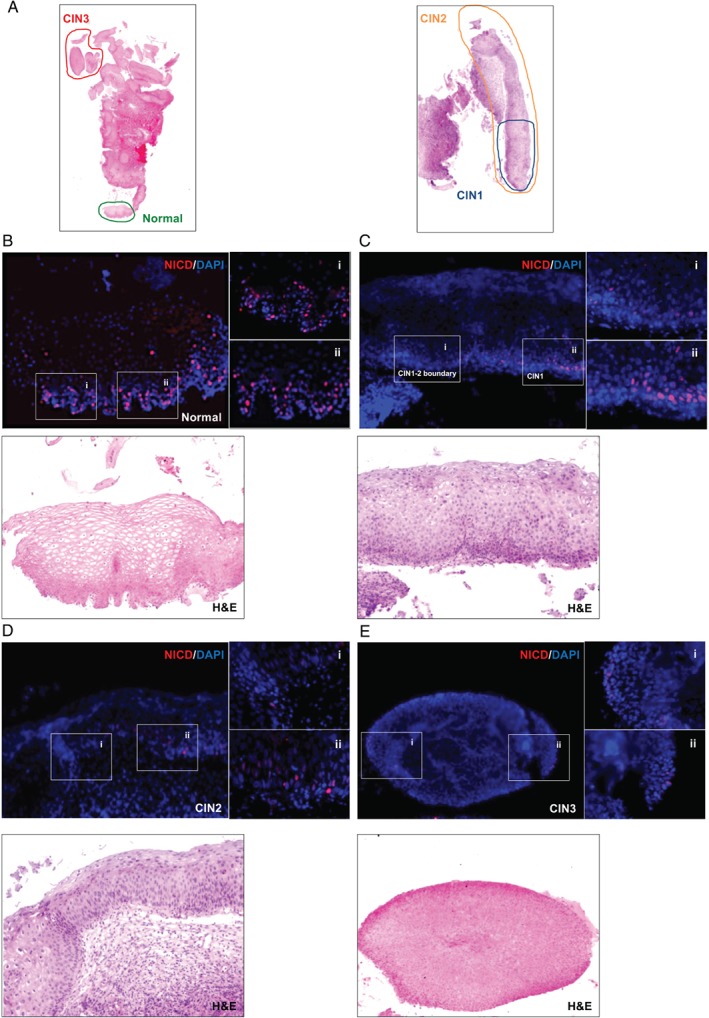
Loss of NICD expression correlates with abortive infections in the human cervix. (A) Low‐power images of H&E‐stained HPV‐16‐positive cervical lesions. Cervical tissue sections were stained with NICD antibodies followed by TMR tyramide fluorescence signal amplification. Tissue sections were counterstained with DAPI. Digital images of stained sections were acquired with a Pannoramic Slide Scanner prior to counterstaining with H&E. Coloured circles with relative CIN grading (according to pathologist's diagnosis) mark the areas shown in detail in panels B–E. (B–E) Magnified images showing the pattern of NICD staining and relative H&E counterstains in normal cervix (B), CIN1 (C), CIN2 (D), and CIN3 (E) (see also supplementary material, Figure S10).

In order to confirm that comparable Notch1 activation was similarly affected during in vivo infection, NICD was examined in characterized patient‐derived CIN1–3, as well as uninfected cervix. Representative NICD patterns were collected from tissue areas where three pathologists independently agreed on the neoplastic grade (Figure 5A). As seen in raft tissue, patient‐derived normal cervical squamous epithelium showed prominent NICD staining in the immediate parabasal layers and in scattered cells of the basal layer (Figure 5B; 5Bi, ii; and supplementary material, Figure S8). Adjacent to this uninfected epithelium, an area of productive infection was apparent using cell cycle (MCM2) and viral (HPV‐16 E4) biomarkers 9, 52 (supplementary material, Figure S10). In this region, cleaved Notch was absent in the basal layer but was maintained in parabasal cells (Figure 5B; see also Figure 4). In low‐grade (CIN1/LSIL) lesions, a strong positivity for NICD could still be observed in the lower parabasal layers as well as in basal cells (Figure 5 Ci and supplementary material, Figure S8). By contrast, the transition towards transforming infection (CIN2/HSIL; supplementary material, Figure S10) was characterized by a general reduction in the intensity of staining and the number of NICD‐positive basal and parabasal cells (Figure 5Cii; Di, ii; and supplementary material, Figure S8), which was more evident in areas of higher‐grade disease (CIN3/HSIL; Figure 5Ei, ii and supplementary material, Figures S8 and S10).

## Discussion

In this study, we have examined Notch1 disruption by E6 in productive infection and during abortive/transforming infection. We provide evidence that in productive infections, the activity of full‐length E6 is subjected to a tight restriction in the basal layer, likely through the modulation of its splicing patterns, and that the progression to high‐grade lesions is associated with an expansion of the cell population able to support the activity of E6.

In the uninfected epithelium and in rafts, Notch cleavage is first seen in the basal layer, but increases in the parabasal layers, coinciding closely with the first induction of keratin‐10. This pattern is typical of squamous epithelia 53 and is consistent with the enrichment of Notch receptors and ligands (Dll1 and Jag2) in these epithelial layers 54. A similar pattern of expression was also observed for p53, which fits well with the role of p53 as a Notch transcriptional activator 37, 41.

**Figure 6:**
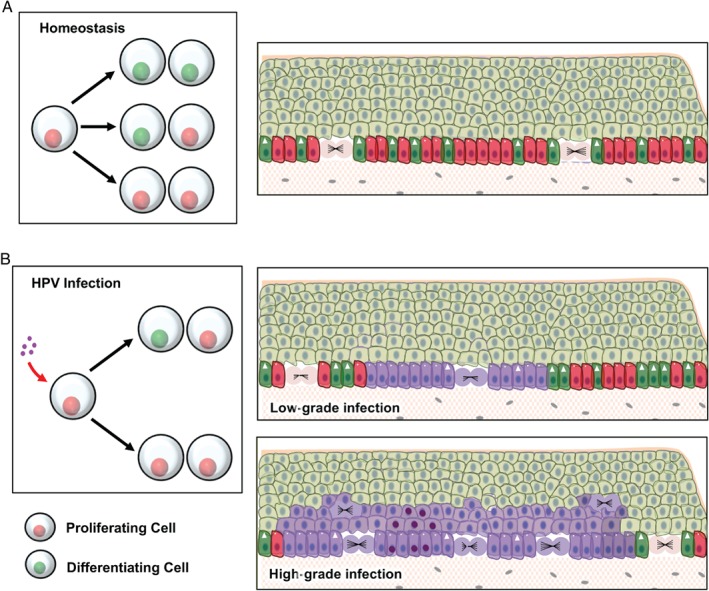
Proposed model for the modulation of keratinocyte cell fate based on the inactivation of p53 and Notch by E6 expression. (A) Homeostasis in the squamous epithelium is determined by the balanced probability of the outcome of each cell division: two differentiating cells, one differentiating and one proliferating progenitor, and two proliferating progenitors (left panel). According to this model, the fate of each division is stochastic; however, across the total population, the odds of having one of the three possible outcomes is balanced. As a result, in the basal layer the proliferation of progenitor cells (red cells with circles) compensates for the loss of cells by terminal differentiation (green cells with arrowhead). (B) Upon infection with HPV‐16, the inactivation of p53 and Notch leads to an unbalanced fate of cell divisions, with a skew towards proliferation (left panel). In low‐grade lesions (upper‐right panel), reduced levels of p53 and Notch in HPV‐infected (purple) cells allow for the maintenance and expansion of the HPV‐infected pool in the basal layer. However, low levels of E6 and E7 expression allow for an adequate level of keratinocyte differentiation able to sustain the viral life cycle. Its deregulation (lower‐right panel) is thought to be associated with an elevation of E6 and E7 expression. Increased E6 levels further reduce the proportion of differentiating cells within the infected basal cell population, allowing for their clonal expansion and persistence with the colonization of large areas of the epithelium. Increased levels of proliferation and long‐term persistence in the epithelium may eventually lead to the accumulation of oncogenic mutations (cells with dark purple nuclei) predisposing to the development of malignancy.

A recent study has suggested a role for Notch in progenitor keratinocytes, where it is required for the maintenance of an undifferentiated stem cell‐like phenotype 55. Other studies have shown that Notch activity represents the switch promoting commitment to differentiation in mouse embryonic keratinocytes 56, suggesting that Notch activation identifies progenitor keratinocytes that are committed to differentiation. This is also supported by promoter activity studies, which reveal that the differentiation marker involucrin can be detected in occasional basal keratinocytes in murine interfollicular skin epidermis 42. Again, these cells are regarded as progenitors that are committed to leave the epithelial basal layer and undergo terminal differentiation. Differentiation markers can also be detected in human keratinocytes grown in monolayer culture, with committed cells being eventually excluded from the cell monolayer to allow their differentiation 57. When taken together, these results invoke a competition between proliferating and differentiating cells in the epithelial basal layer, and depict the stratification of differentiating keratinocytes as the consequence of fate commitment towards terminal differentiation rather than the cause 57 (Figure 6A). This paradigm fits well with the detection of cleaved Notch in basal/parabasal keratinocytes prior to the first appearance of keratin‐10. This is further supported by the FACS sorting of differentiating NIKS populations, which similarly shows that the activation of Notch, and the detection of early events during keratinocyte differentiation (as marked by increased keratin‐10 expression), is also temporally segregated in monolayer culture.

Interestingly, the E6‐mediated down‐regulation of the Notch pathway is not an exclusive function of high‐risk HPV types, with recent studies suggesting that β‐HPVs can also inhibit Notch signalling, albeit through a different mechanism 58, 59, 60. Both E6 proteins interact with a short LXXLL amino acid motif that is present in both the ubiquitin ligase E6AP (a high‐risk E6 interactor) and the Notch transcriptional co‐activator MAML1 (a beta HPV interactor). The role of E6AP in p53 depletion is well established, with both alpha and beta HPV types disrupting normal Notch signalling to some extent 58, 59. These observations suggest that the inhibition of Notch and the modulation of keratinocyte differentiation are important for a wide range of HPV pathologies.

Our analysis of LSIL‐like HPV‐16 raft culture indicates that in productive infections, the degradation of p53 and the down‐regulation of NICD are restricted to the basal layer of the epithelium, an effect that, to our knowledge, has not been previously described. The activation of the Notch pathway is a switch governing the transition from keratinocyte proliferation to differentiation 31. The restricted inactivation of Notch signalling in the basal layer provides a mechanistic insight into the way that the high‐risk α‐HPV E6 proteins can contribute to basal cell persistence and expansion 3. At the same time, the virus must ensure adequate levels of differentiation to support viral genome amplification. In our study and others 29, it appears that signal transduction from growth factor receptors, such as EGFR, plays a critical role in modulating HPV‐16 E6 function by restricting full‐length E6 expression to a growth factor‐rich environment. In stratified epithelia, this environment occurs in the lower epithelial layers, which are subject to dermal growth factor stimulation and which express EGFR 61. It appears that such a mechanism might represent an evolutionary adaptation of the high‐risk mucosal HPV types to limit the extent to which their full‐length E6 proteins are expressed in the differentiating layers. This is also consistent with the fact that in HSIL‐like HPV‐16 NIKS rafts, the levels of basal and parabasal p53 and NICD are dramatically reduced compared with LSIL‐like and parental NIKS raft cultures. This correlates with a lower level of Krt10 induction, supporting a crucial role for the elevation of E6 and E7 activity in the deregulation of the HPV life cycle 4, 27.

Keratinocyte squamous differentiation is an extremely complex biological process, involving major changes in the pattern of gene expression, morphology, and cell function. The combination of these changes leads basal keratinocytes to form a stratified tissue formed of three layers: spinous, granular, and cornified. Terminally differentiated cells in the stratum corneum (i.e. the cornified layer) are subject to nuclear degeneration and form a continuous barrier that is mechanically strengthened by the cross‐linking of keratin filaments 5. The initial event triggering differentiation in basal keratinocytes is thought to lead to asymmetrical cell division and the migration of committed daughter cells into the parabasal epithelial layers. One of the crucial consequences of this transition is reduced exposure to proliferative cues and more restricted expression of molecules involved in cell proliferation [e.g. integrins and receptor‐associated tyrosine kinases (RTKs)]. Keratinocyte differentiation therefore results from the balanced activity of proliferative and anti‐proliferative pathways, including RAS–MAPK, TGFβ, TGFα, integrins, RTKs, Notch, p63, and others, which have been reviewed elsewhere 62, 63, 64.

While trying to avoid any oversimplification of HPV‐16 E6 function and its effect on keratinocyte differentiation, we feel that the data presented here highlight a physiologically very important role of E6 in modulating initial cell fate decisions in basal keratinocytes, through the inhibition of Notch signalling. Such a potent fate‐determining effect has been hypothesized to occur when asymmetric cell divisions lead to the inheritance of a stronger Notch signal by one of the daughter cells, thereby counteracting proliferative signals and promoting differentiation 65, 66. This scenario is also supported by recent studies in mice, where the accumulation of inactivating mutations in *Tp53* and *Notch* genes confers a so‐called ‘super‐competitor’ phenotype, skewing keratinocyte cell fate towards proliferation rather than differentiation 13, 21. This allows the persistence and clonal expansion of mutant cell populations, and similar mechanisms of clonal persistence have been recently described in sun‐exposed human skin 12. It is therefore tempting to speculate that HPV‐16 E6, through the coupled inactivation of both p53 and Notch, might confer a similar competitive phenotype to infected cells (Figure 6B), a condition particularly relevant in abortive infections, as well as HPV‐driven cancers where E6 levels are thought to increase 4 (Figure 6B). Such an interpretation is again supported by our FACS sorting experiments, which clearly showed that the expression of HPV‐16 E6 interferes with activation of the Notch pathway prior to the induction of differentiation (Krt10‐dim population). In addition, and in contrast to E7, our 2D monolayer experiments highlight E6 as a major stimulator of cell growth, particularly at high cell densities. In this situation, the expression of HPV‐16 E6 was necessary and sufficient to (i) recapitulate the growth advantage phenotype of HSIL‐like NIKS, (ii) abrogate the expression of full‐length and cleaved Notch1, and (iii) prevent induction of Krt10 in post‐confluent NIKS monolayers. Consistent with a scenario in which the E6‐mediated degradation of p53 contributes to the acquisition of these neoplastic traits, the transient ablation of p53 led to a reduced level of *Notch* mRNA and protein expression and to an increased proliferation rate of NIKS cells. It is interesting to note that the transient ablation of the p53 family member p63 in NIKS led to the opposite phenotype. Nonetheless, this is consistent with the fact that p63 is up‐regulated in HPV‐driven and HPV‐negative squamous cell carcinomas 67, 68, 69, 70, 71, suggesting a role in supporting keratinocyte proliferation 38. In our LSIL‐ and HSIL‐like HPV‐16 NIKS raft cultures, p63 levels were progressively up‐regulated in the differentiating layers of the epithelium, along with the marker for cell cycle entry MCM7. Interestingly, levels of ΔNp63, the p63 isoform responsible for antagonizing p53 activity and driving cell proliferation 72, were more strongly up‐regulated in the 5H NIKS HPV‐16 raft culture, which was also the most disrupted with regard to pattern of p53, NICD, and Krt10 expression.

When taken together, our study highlights the importance of E6 and E7 elevation during the progression from productive to abortive (i.e. transforming) HPV infections. The modulatory effects of E6 on keratinocyte cell fate have important implications on the clonal expansion of infected cells and their persistence in the epithelium, ultimately favouring the onset of malignancy.

## Author contributions statement

JD, CK, and CU planned the experiments. CU, CK, and DL conducted the experiments. EI generated episomal HPV‐16 NIKS cell lines and organotypic rafts. HG coordinated the clinical studies and performed the staining on clinical material. RM processed histological samples. CK and JD wrote the paper.

**SUPPLEMENTARY MATERIAL ONLINE**

**Supplementary materials and methods**

**Supplementary figure legends**

**Figure S1.** HPV 16E6 decreases the doubling time of NIKS cells

**Figure S2.** HSIL‐like NIKS display increased growth advantage compared with LSIL‐like cells

**Figure S3.** EGF signalling controls the splicing pattern of E6 from the full‐length HPV‐16 genome

**Figure S4.** Determination of optimal keratin‐10 antibody concentration for FACS analysis

**Figure S5.** The ablation of p53 and of p63 has opposing effects on NIKS proliferation

**Figure S6.** Histological and molecular verification of episomal HPV‐16 rafts and LXSN HPV‐16 E6 and E7 rafts

**Figure S7.** Expression of NICD, p53, and keratin‐10 in the lower layers of NIKS, LSIL‐like, and HSIL‐like NIKS rafts

**Figure S8.** Quantification of the protein expression patterns in the raft epithelium

**Figure S9.** Correlation of the differentiation status of HPV‐16 NIKS and LXSN 16E6 and 16E7 raft cultures with the expression of markers for cell cycle entry and the basal layer

**Figure S10.** Association of cervical phenotype with molecular markers of cell cycle entry and HPV life cycle

## Supporting information


**Supplementary materials and methods**

**Supplementary figure legends**

**Figure S1.** HPV 16E6 decreases the doubling time of NIKS cells. (A) Doubling times were calculated using a non‐linear regression curve fit according to the total cell numbers at the beginning and at the end of each growth assay indicated in Figure 1. The mean values with ± SD are shown. (B) The left panel compares the cell numbers measured from the point of confluence onwards (∼1 × 10^6^ cells, red dotted line) in the growth assay in Figure 1A. The right panel represents the cell numbers measured from day 5 of 9 days' growth assays with no added serum or EGF. In both cases, the expression of HPV‐16 confers a significant growth advantage to NIKS cells. Bars represent median values.

**Figure S2.** HSIL‐like NIKS display increased growth advantage compared with LSIL‐like cells. (A) Equal numbers of NIKS, NIKS 2L, and NIKS 4H HPV‐16 lines were seeded into six‐well plates and grown for a total of 9 days before harvesting and counting. Each plotted point of the growth assay represents the average total cell number per well counted at each time point (days 1, 3, 5, 7, and 9). Error bars represent ± SD (n = 3). The plot on the right‐hand side represents doubling times calculated with the cell numbers obtained in the growth assays in panel A. (B) Representative bright‐field images show the differences in cell density among the cell lines used in panel A at days 3 (subconfluent), 5 (confluent), and 7 (post‐confluent). (C) The pattern of filaggrin expression was assessed by immunofluorescence analysis of individual NIKS, NIKS 2L, and 4H raft culture sections using Alexa594‐conjugated secondary antibodies. All sections were counterstained with DAPI.

**Figure S3.** EGF signalling controls the splicing pattern of E6 from the full‐length HPV‐16 genome. (A) Organization of the bicistronic HPV16 E6/E7 pre‐mRNA. Base pair numbers showing the position of E6 and E7 genes relative to the HPV‐16 genome. Exclusion of exons 226–409 results in the formation of the E6* ORF. Arrows indicate primer localization for semi‐quantitative RT‐PCR. (B) Semi‐quantitative comparative RT‐PCR showing the expression of full‐length (343 base pairs) and spliced HPV‐16 E6 (161 base pairs) in NIKS HPV16 cells with increasing concentrations of EGF (10, 100, 500 ng/ml from left to right). GAPDH was used as a loading control.

**Figure S4.** Determination of optimal keratin‐10 antibody concentration for FACS analysis. (A, B) NIKS cells grown to post‐confluence were recovered by trypsinization followed by fixation and permeabilization as detailed in the Material and methods section. Cells were then incubated with the indicated concentrations of primary antibody, followed by incubation with Alexa 488‐conjugated secondary antibody and FACS sorting of Krt10‐bright and ‐dim populations. (C, D) Post‐confluent NIKS cells were treated as in panel A, with the exception that they were incubated with increasing concentration of isotype control (IgG1) control antibody.

**Figure S5.** The ablation of p53 and of p63 has opposing effects on NIKS proliferation. (A) NIKS cells were seeded, transfected with the indicated RNAi oligonucleotides, and left to grow for a total of 5 days prior to harvesting and counting. The average total cell number was plotted against each time point assayed (days 1, 3, and 5). Each point represents the average result from three independent experiments. Error bars represent ± SD. (B) Representative bright‐field pictures show the differences in cell density obtained at each time point of the growth assay in panel A. (C) Total cell extracts were prepared from cells harvested at day 5 of the growth assay in panel A. The patterns of expression of the indicated proteins were assessed by western blot using GAPDH as a protein loading control.

**Figure S6.** Histological and molecular verification of episomal HPV‐16 rafts and LXSN HPV‐16 E6 and E7 rafts. (A) Haematoxylin and eosin‐stained sections of raft cultures prepared from NIKS or NIKS HPV‐16 clonal lines analysed in Figure 4. (B) Expression of the HPV‐16 life cycle‐associated proteins E1^E4 and L1 were used to evaluate the life cycle status (productive or abortive) in raft cultures prepared from HPV‐16 episomal lines.

**Figure S7.** Expression of NICD, p53, and keratin‐10 in the lower layers of NIKS, LSIL‐like, and HSIL‐like NIKS rafts. Images of individual raft cultures stained as detailed in Figure 4 were acquired at higher magnification (40×) to show differences in the appearance of p53, NICD, and keratin‐10 in the lower epithelial layers of normal and HPV‐16 NIKS raft cultures.

**Figure S8.** Quantification of the protein expression patterns in the raft epithelium. For each of the indicated phenotypes, the surface area of the raft or cervical epithelium showing positive staining for each indicated protein was quantified and plotted as a percentage of the total raft area. Surface areas were extrapolated based on the number of pixels of each image.

**Figure S9.** Correlation of the differentiation status of HPV‐16 NIKS and LXSN 16E6 and 16E7 raft cultures with the expression of markers for cell cycle entry and the basal layer. (A) Representative images of the pattern of MCM7, p63, and ΔNp63 expression in raft cultures of NIKS or NIKS HPV‐16 episomal lines as in panels A–C. The fluorescence signal for MCM7, p63, and ΔNp63 was amplified with TMR. All sections were counterstained with DAPI. (B) Representative images showing the expression of NICD and Krt10 or MCM7 in raft cultures prepared from control (LXSN) NIKS or NIKS expressing either HPV‐16 E6 or E7. All sections were counterstained with DAPI. (C) Haematoxylin and eosin‐stained sections of raft cultures prepared from NIKS LXSN or NIKS expressing either HPV‐16 E6 or E7 analysed in panel B.

**Figure S10.** Association of cervical phenotype with molecular markers of cell cycle entry and HPV life cycle. The CIN grading of individual cervical tissue sections described in Figure 6 was correlated with the expression of the marker for cell cycle activity MCM2 (red) and the marker of life cycle status HPV E1^E4 (green). All sections were counterstained with DAPI.
