# Supplementary material for: Modulation of basal cell fate during productive and transforming HPV‐16 infection is mediated by progressive E6‐driven depletion of Notch
Source: J Pathol. 2017 Jul 24;242(4):448–62. doi: 10.1002/path.4917 (PMC5601300; doi:10.1002/path.4917)
Supplement: Supplementary file 1 — Supplementary materials and methods [file PATH-242-448-s006.docx]

**Supplementary materials and methods**

Reference numbers refer to the main text list

*Plasmids*

The plasmid pSPW12 containing the HPV16 genome was a kind gift from Professor Margaret Stanley (Department of Pathology, University of Cambridge, Cambridge, UK) [25]. pMV11 and pMV11_HPV-16 E6 constructs have been described previously [73]. Retroviral vectors pLXSN, pLXSN HPV16 E6, pLXSN HPV16 E7, and pLXSN HPV16 E6/E7 were kindly provided by Dr Denise Galloway (Human Biology Division, Fred Hutchinson Cancer Research Center, Seattle, WA, USA) [74]. For the generation of HPV-16 E6 mutants, wt E6 was mutated using PfuUltra (Stratagene, London, UK) and the following primer pairs: E6ΔPBMforward: 5'gcagatcatcaagaacacgtagataaacccagctgtaatcatgcatgg3'; E6ΔPBMreverse: 5'gcagctctgtgcataactgtggtaactttgtggcgctctcctgtgggtcctgaaacattgc;

E6SATforward: 5'GCAATGTTTCAGGACCCACAGGAGAGCGCCACAAAGTTACCACAGTTATGCACAGAGCTGC3';

E6SATreverse: 5'GCAGCTCTGTGCATAACTGTGGTAACTTTGTGGCGCTCTCCTGTGGGTCCTGAAACATTGC3'.

*Cell culture and transfection*

293TT and J2-3T3 mouse fibroblasts were maintained in Dulbecco’s modified Eagle’s medium (DMEM; Sigma, Haverhill, UK) supplemented with 10% fetal bovine serum, penicillin–streptomycin (100 U/ml), and glutamine (300 μg/ml). For the generation of feeder cells, confluent J2-3T3 cells were harvested by trypsinization and irradiated at 60 Gy using an X-ray generator. NIKS keratinocytes were a kind gift from Professor Paul F Lambert (McArdle Laboratory for Cancer Research, University of Wisconsin, Madison, WI, USA). NIKS keratinocytes were maintained at sub-confluent levels on gamma-irradiated J2-3T3 feeder cells in complete F medium with all the supplements as previously described [28]. To harvest NIKS, the cells were washed with 8–10 ml of PBS and then incubated with 1 ml of trypsin–versene for 2 min at 37°C in order to remove the fibroblast layer. The enzyme solution was then aspirated and keratinocytes were incubated in 2 ml of fresh trypsin–versene at 37°C for an additional 5–10 min or until cells became lifted. Differentiation of NIKS and NIKS-derived cell lines in organotypic raft culture was performed essentially as described previously [75]. For the generation of NIKS cells carrying episomal HPV-16 genomes, cells were co-transfected with recircularized replication-competent W12-HPV-16 genomes and a pCDNA6 (Invitrogen, Paisley, UK) vector that contained a blasticidin (A1113903; Life Technology, Paisley, UK) resistance gene using Effectene (Qiagen, Manchester, UK) according to the manufacturer’s instructions. Clonal cell lines were recovered after several days of blasticidin (A1113903; Life Technology, Paisley, UK) selection (6 µg/ml) and were individually expanded into six-well plates after the individual colonies became visible. The presence of episomal copies of the HPV-16 genome was assessed by Southern blot [27].

For the delivery of siRNAs against p63, p53, and luciferase (Dharmacon, Lafayette, CO, USA) in HPV-negative NIKS cells, cells were seeded on six-well plates at a density of 1 × 10^5^ on 1 × 10^5^ γ-irradiated feeders and were transfected using Lipofectamine RNAiMax (Life Technologies, Paisley, UK) according to the manufacturer’s instructions. Depending on the experimental setting, cells were harvested 48, 72, or 96 h after transfection. For the delivery of siRNA against luciferase or HPV-16 E6/E7 (GAGCUGCAAACAACUAUA) in experiments involving NIKS HPV-16 clonal lines, 1.5 × 10^6^ cells were electroporated with 100 nm siRNA using the 4D Nucleofector X Unit (Lonza, Zurich, Switzerland) and Amaxa SE Cell Line 4D-Nucleofecto X Kit L (Lonza), according to the manufacturer’s instructions. Subsequently, cells were seeded into six-well plates on top of 1 × 10^5^ feeder cells in complete F medium and given fresh medium the next day. Cells were harvested and counted 24, 48, 72, and 96 h after siRNA transfection.

For the generation of stable NIKS cell lines expressing LXSN, LXSN HPV-16 E6, E7 or E6/E7, pseudo-typed retroviral particles were generated from 293TT using standard protocols. 293TT cells were transfected using the TransIT-293 transfection reagent (Mirus, Madison, WI, USA) according to the manufacturer’s instructions. After infection, NIKS cells were selected with G418 (100 μg/ml) for several days before experimental use.

*Antibodies*

The antibodies used in this study for western blot were cleaved Notch1 Val1744 (D3B8; Cell Signaling Technology, Columbus, OH, USA), Notch1 (D1E11; Cell Signaling Technology), p21 (05-345; Millipore, Watford, UK), pRb (554136; BD Pharmingen), HES1 (D6P2U; Cell Signaling Technology), Krt10 (PA5-32459; Thermo Scientific, East Grinstead, UK), and GAPDH (MA B374; Millipore). For immunofluorescence and immunohistochemistry, the antibodies were cleaved Notch1 Val1744 (D3B8; Cell Signaling Technology), p53 (DO-1; Santa Cruz Biotechnology, Heidelberg, Germany), Krt10 (PA5-32459; Thermo Scientific), MCM7 (ab52489; Abcam, Cambridge, UK), MCM2 (ab4461; Abcam), pan-p63 (ab735; Abcam), ΔNp63 (poly6190; Biolegend, London, UK), and HPV-16 L1 (K1H8; Dako, London, UK).

*Immunofluorescence and immunohistochemistry*

Raft sectioning, dewaxing, rehydration, and blocking were carried out as described previously [76]. For all raft sections, epitope exposure was carried out in citrate buffer (pH 6) at 110 °C for 15 min, whereas sections from clinical samples were treated as described previously [9]. For the detection of NICD, MCM7, p63, and HPV-16 L1 slides were incubated with antibodies overnight at 4 °C in a humidified chamber; Krt10, p53, and ΔNp63 antibodies were incubated for 1 h at room temperature. The detection of MCM2 and HPV-16 E4 in cervical biopsies has been described previously [9]. Nuclear counterstaining was performed with 4',6-diamidino-2-phenylindole (DAPI; Sigma, Haverhill, UK). Unless otherwise stated, primary antibodies were detected using an HRP polymer detection system (Envision G2 Doublestain System; Dako, London, UK) followed by fluorescence development with tetramethyl rhodamine (TMR) tyramide solution (TSA Fluorescence Systems; Perkin Elmer, Beaconsfield, UK). For HPV-positive cervical biopsies, the detection of NICD antibody was performed using an ImmPRESS HRP Anti-rabbit IgG Polymer Detection Kit (Vector Laboratories, Philadelphia, PA, USA). Labelling of the Krt10 antibody was carried out using a Zenon Alexa Fluor 647 Mouse IgG1 Labelling Kit according to the manufacturer’s instructions. HPV-16 16 E4 was detected with clone TVG405 Fab [77] directly conjugated to Alexa Fluor 488. Fluorescence images were acquired using a Zeiss Axiovert A1 fluorescence microscope or a Pannoramic Slide Scanner (3D Histotech, Budapest, Hungary). After fluorescence acquisition, all sections were H&E stained and images were taken under bright-field illumination.

For the immunofluorescence analysis of Krt10 expression in cells grown in 2D monolayer, NIKS and derived lines were seeded as described for growth assays on glass coverslips in six-well plates. Cells were left to grow for 3, 5 or 7 days and at each time point, they were fixed in 4% paraformaldehyde and permeabilized with 0.1% Triton X-100 in PBS. The antibody was incubated for 1 h at room temperature, followed by incubation with anti-mouse Alexa 594 conjugated antibody. Nuclear counterstaining was performed with DAPI (Sigma). Secondary antibodies conjugated with Alexa Fluor dyes were purchased from Life Technologies (Paisley, UK).

*RT-PCR and quantitative PCR (qPCR)*

Total RNA extraction was carried out using RNeasy Mini Kits (Qiagen, Manchester, UK) according to the manufacturer’s protocol. Genomic DNA was removed from total RNA samples using the DNA-free kit (Ambion, Loughborough, UK) according to the manufacturer’s protocol, and RNA was either stored at −80°C or used immediately in RT-PCR reactions. Reverse transcription reactions were performed using SuperScript First-Strand Synthesis Kits (Life Technologies) and oligo(dT) primers according to the manufacturer’s instructions. qPCR was performed using the Viia 7 real-time PCR system (Applied Biosystems, Loughborough, UK) with SYBR Green PCR master mix (Applied Biosystems). In brief, 2 μl of cDNA was added to 32 μl of SYBR Green PCR master mix, 6.4 μl of 10 μm primers, and ddH_2_O to a final volume of 64 μl, and 20 μl of the reaction was dispensed in triplicate in 384-well plates (Applied Biosystems). The amplification reaction was pre-degenerated for 15 s at 95.0°C, followed by 45 cycles of 95.0°C for 15 s and 60.0°C for 1 min. Specific primer sets for *NOTCH1*, *P21*, and *GAPDH* genes were from Origene and for each of the genes analysed, a fragment of DNA corresponding to the region flanked by the primer pair was synthesized (Cambridge Bioscience, Cambridge, UK) and cloned into pQCminusMCS vectors (Blue Heron, Bothell, WA, USA).

The semi-quantitative RT-PCR analysis of the expression of E6 was performed on cDNA samples synthesized from total RNA extracted from NIKS HPV-16 lines as described above. Primer sequences for the amplification of full-length E6 and E6* have been described previously [29].

*Western blot analysis*

Cell pellets were resuspended in 25 U of benzonase (Invitrogen, Paisley, UK) before lysis in protein extraction buffer (150 mm NaCl, 1% Triton X, 0.5% sodium deoxycholate, 6% SDS, 50 mm Tris, pH 8, and 0.005 mm EDTA, pH 8). Protein samples were quantified using a BCA protein assay kit (Pierce, Loughborough, UK) and separated on 4–12% gradient polyacrylamide–SDS–Tris–Tricine denaturing gels (Invitrogen) and transferred onto PVDF membranes (Bio-Rad, Hemel Hempstead, UK). Membranes were blocked for 1 h at room temperature in 10% milk in Tris-buffered saline (TBS). Blots were then incubated overnight at 4°C with the appropriate antibody diluted in 5% BSA TBS-T (TBS, 0.1% Tween 20). After extensive washing, membranes were incubated with the appropriated IRDye 800CW fluorescent secondary antibody (Licor, Lincoln, NE, USA) for 1 h at room temperature and developed using an Odyssey imaging system (Licor). When required, membranes were stripped and reprobed with a different primary antibody as described previously [78].
