# Supplementary figures and images for: Modulation of basal cell fate during productive and transforming HPV‐16 infection is mediated by progressive E6‐driven depletion of Notch
Source: J Pathol. 2017 Jul 24;242(4):448–62. doi: 10.1002/path.4917 (PMC5601300; doi:10.1002/path.4917)

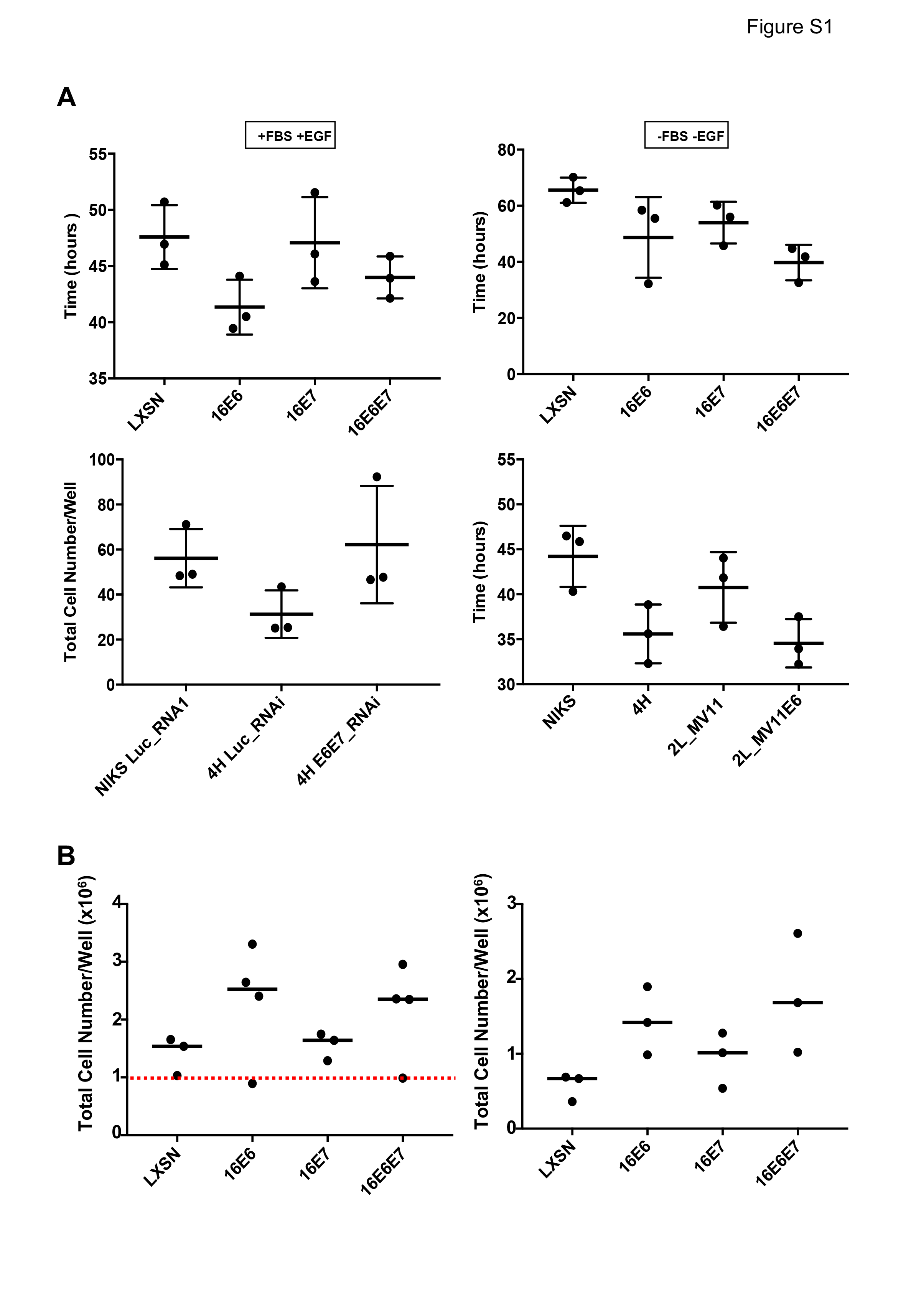

Supplement: Supplementary file 3 — Figure S1. HPV 16E6 decreases the doubling time of NIKS cells. (A) Doubling times were calculated using a non‐linear regression curve fit according to the total cell numbers at the beginning and at the end of each growth assay indicated in Figure 1. The mean values with ± SD are shown. (B) The left panel compares the cell numbers measured from the point of confluence onwards (∼1 × 10^6^ cells, red dotted line) in the growth assay in Figure 1A. The right panel represents the cell numbers measured from day 5 of 9 days' growth assays with no added serum or EGF. In both cases, the expression of HPV‐16 confers a significant growth advantage to NIKS cells. Bars represent median values. [file PATH-242-448-s004.tif]

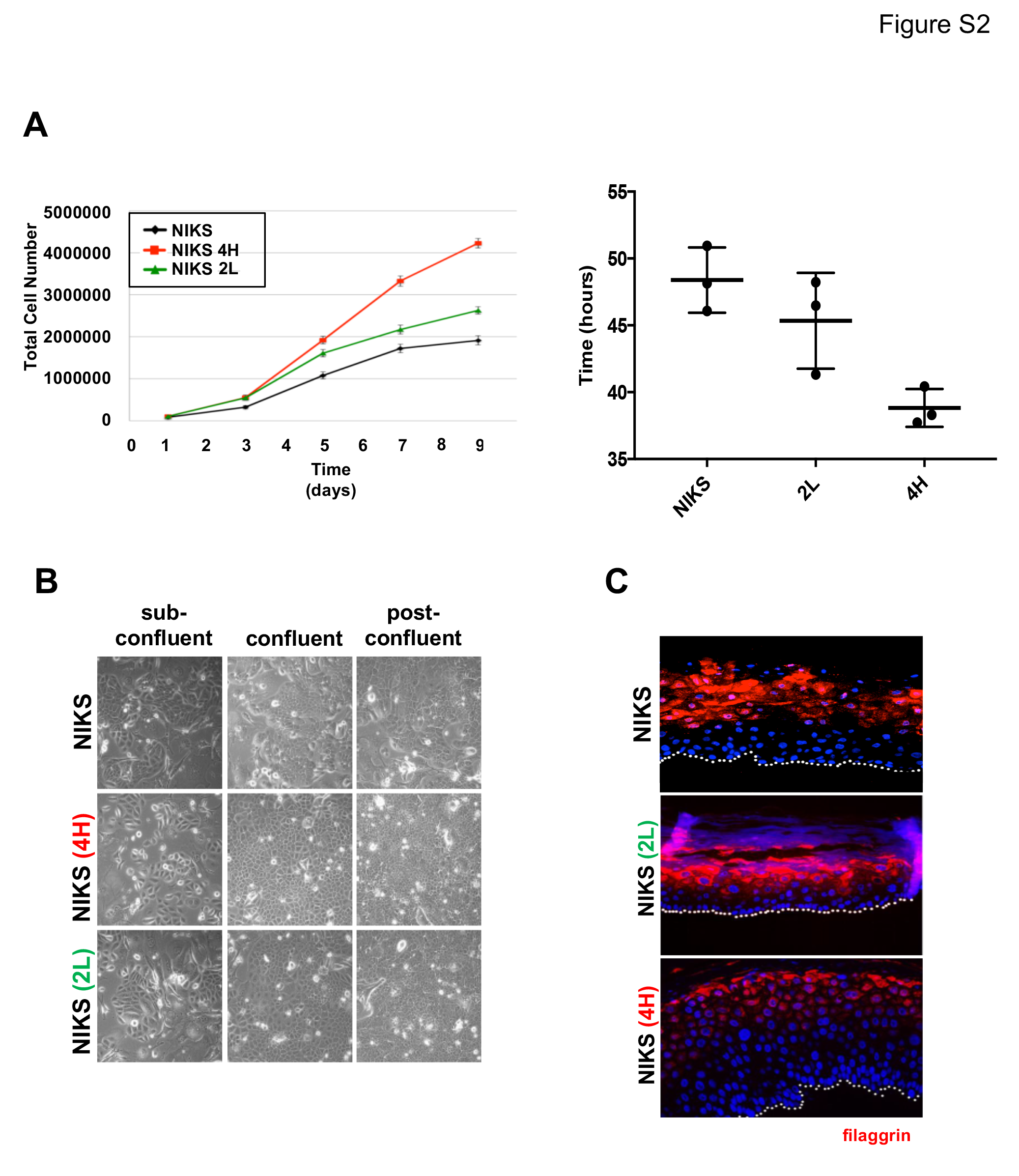

Supplement: Supplementary file 4 — Figure S2. HSIL‐like NIKS display increased growth advantage compared with LSIL‐like cells. (A) Equal numbers of NIKS, NIKS 2L, and NIKS 4H HPV‐16 lines were seeded into six‐well plates and grown for a total of 9 days before harvesting and counting. Each plotted point of the growth assay represents the average total cell number per well counted at each time point (days 1, 3, 5, 7, and 9). Error bars represent ± SD (n = 3). The plot on the right‐hand side represents doubling times calculated with the cell numbers obtained in the growth assays in panel A. (B) Representative bright‐field images show the differences in cell density among the cell lines used in panel A at days 3 (subconfluent), 5 (confluent), and 7 (post‐confluent). (C) The pattern of filaggrin expression was assessed by immunofluorescence analysis of individual NIKS, NIKS 2L, and 4H raft culture sections using Alexa594‐conjugated secondary antibodies. All sections were counterstained with DAPI. [file PATH-242-448-s010.tif]

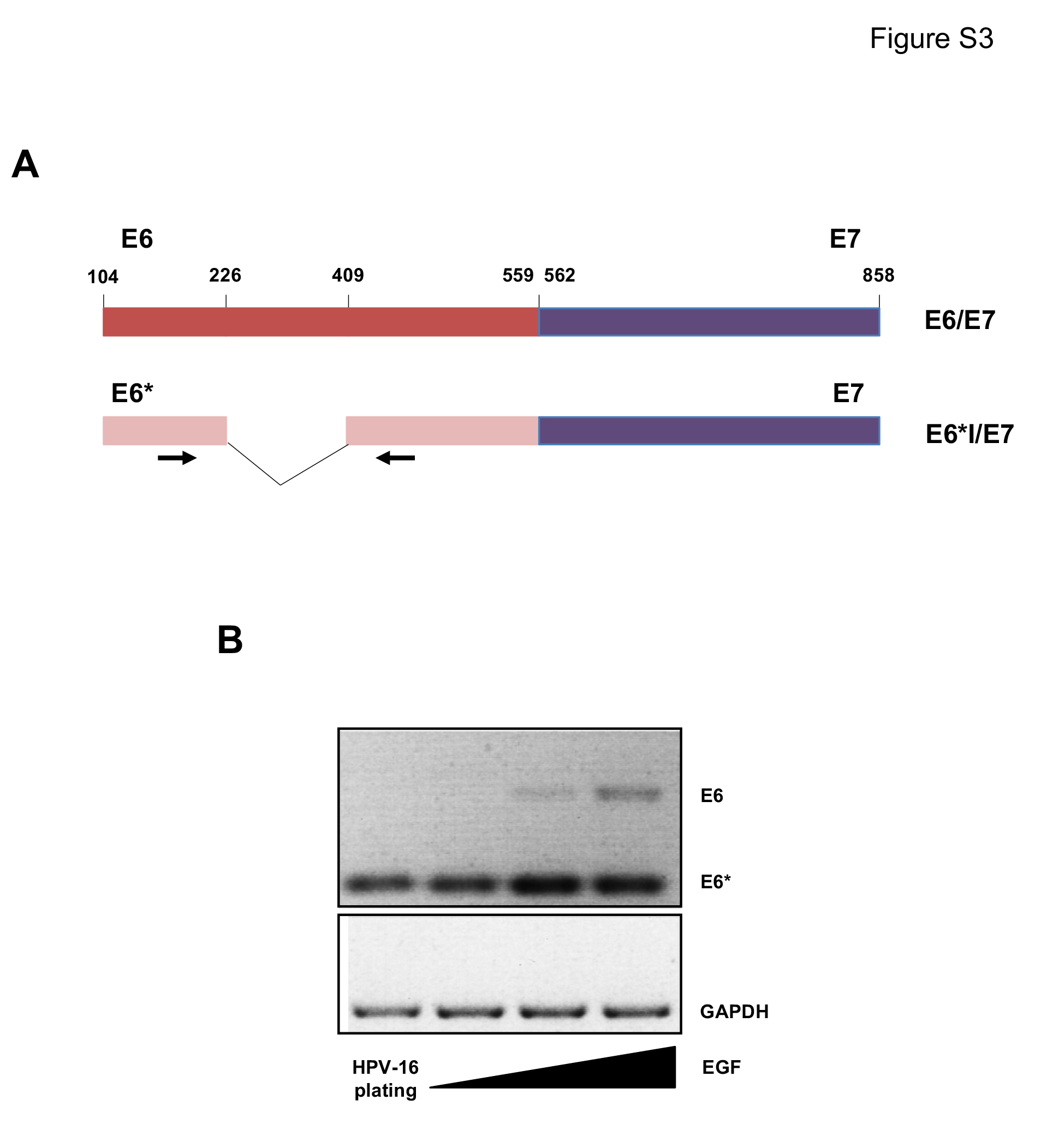

Supplement: Supplementary file 5 — Figure S3. EGF signalling controls the splicing pattern of E6 from the full‐length HPV‐16 genome. (A) Organization of the bicistronic HPV16 E6/E7 pre‐mRNA. Base pair numbers showing the position of E6 and E7 genes relative to the HPV‐16 genome. Exclusion of exons 226–409 results in the formation of the E6* ORF. Arrows indicate primer localization for semi‐quantitative RT‐PCR. (B) Semi‐quantitative comparative RT‐PCR showing the expression of full‐length (343 base pairs) and spliced HPV‐16 E6 (161 base pairs) in NIKS HPV16 cells with increasing concentrations of EGF (10, 100, 500 ng/ml from left to right). GAPDH was used as a loading control. [file PATH-242-448-s003.tif]

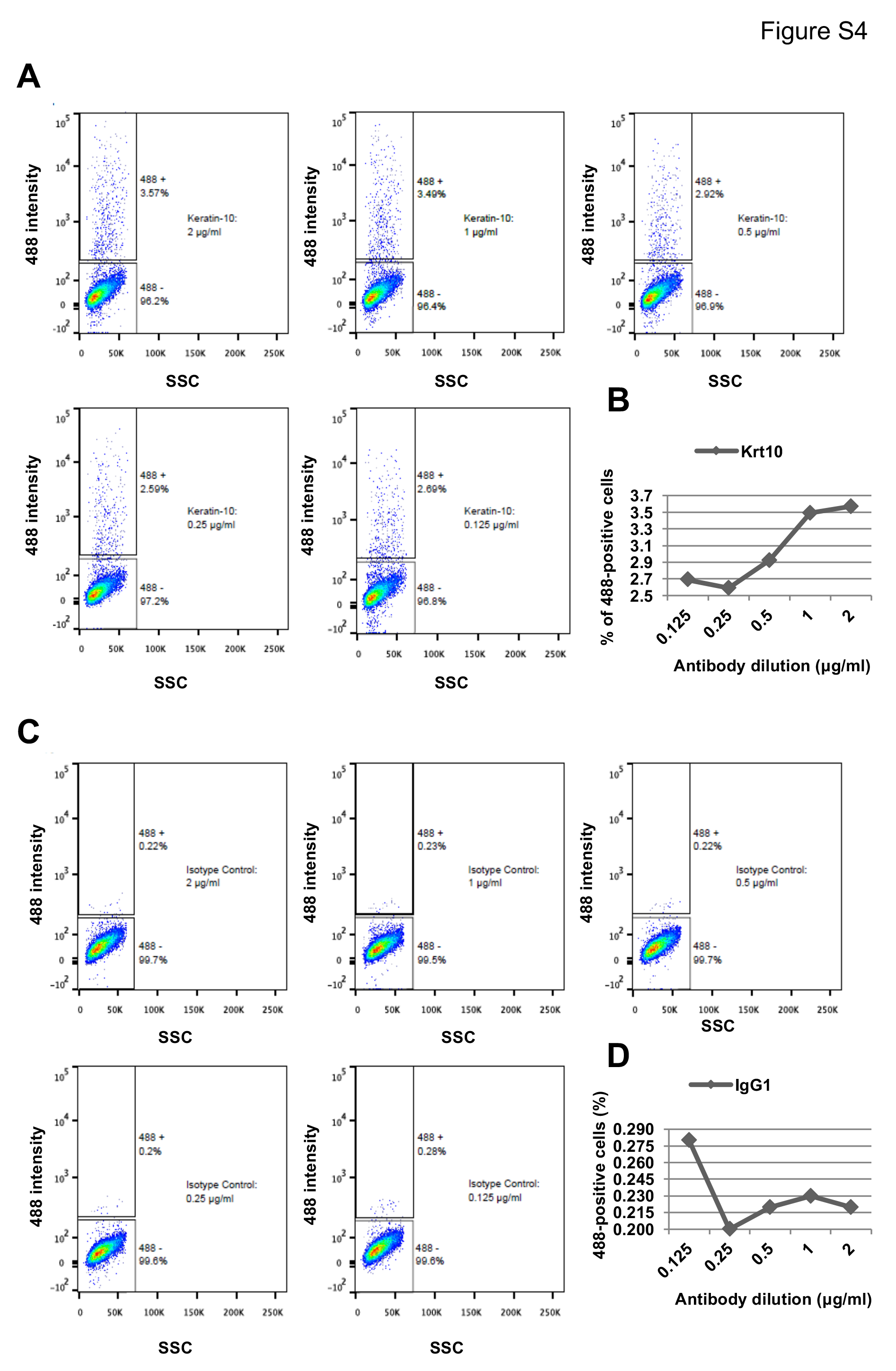

Supplement: Supplementary file 6 — Figure S4. Determination of optimal keratin‐10 antibody concentration for FACS analysis. (A, B) NIKS cells grown to post‐confluence were recovered by trypsinization followed by fixation and permeabilization as detailed in the Material and methods section. Cells were then incubated with the indicated concentrations of primary antibody, followed by incubation with Alexa 488‐conjugated secondary antibody and FACS sorting of Krt10‐bright and ‐dim populations. (C, D) Post‐confluent NIKS cells were treated as in panel A, with the exception that they were incubated with increasing concentration of isotype control (IgG1) control antibody. [file PATH-242-448-s011.tif]

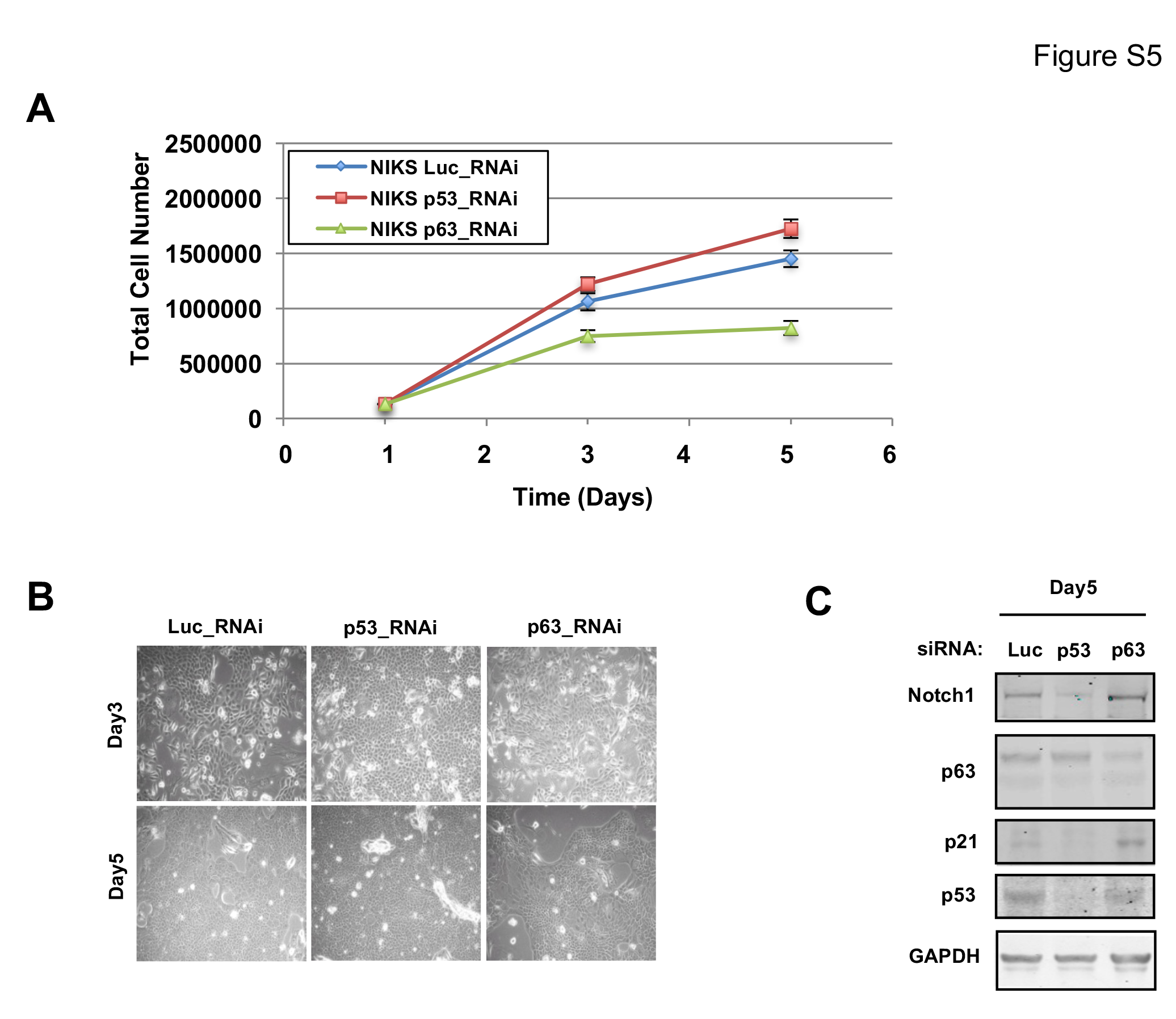

Supplement: Supplementary file 7 — Figure S5. The ablation of p53 and of p63 has opposing effects on NIKS proliferation. (A) NIKS cells were seeded, transfected with the indicated RNAi oligonucleotides, and left to grow for a total of 5 days prior to harvesting and counting. The average total cell number was plotted against each time point assayed (days 1, 3, and 5). Each point represents the average result from three independent experiments. Error bars represent ± SD. (B) Representative bright‐field pictures show the differences in cell density obtained at each time point of the growth assay in panel A. (C) Total cell extracts were prepared from cells harvested at day 5 of the growth assay in panel A. The patterns of expression of the indicated proteins were assessed by western blot using GAPDH as a protein loading control. [file PATH-242-448-s001.tif]

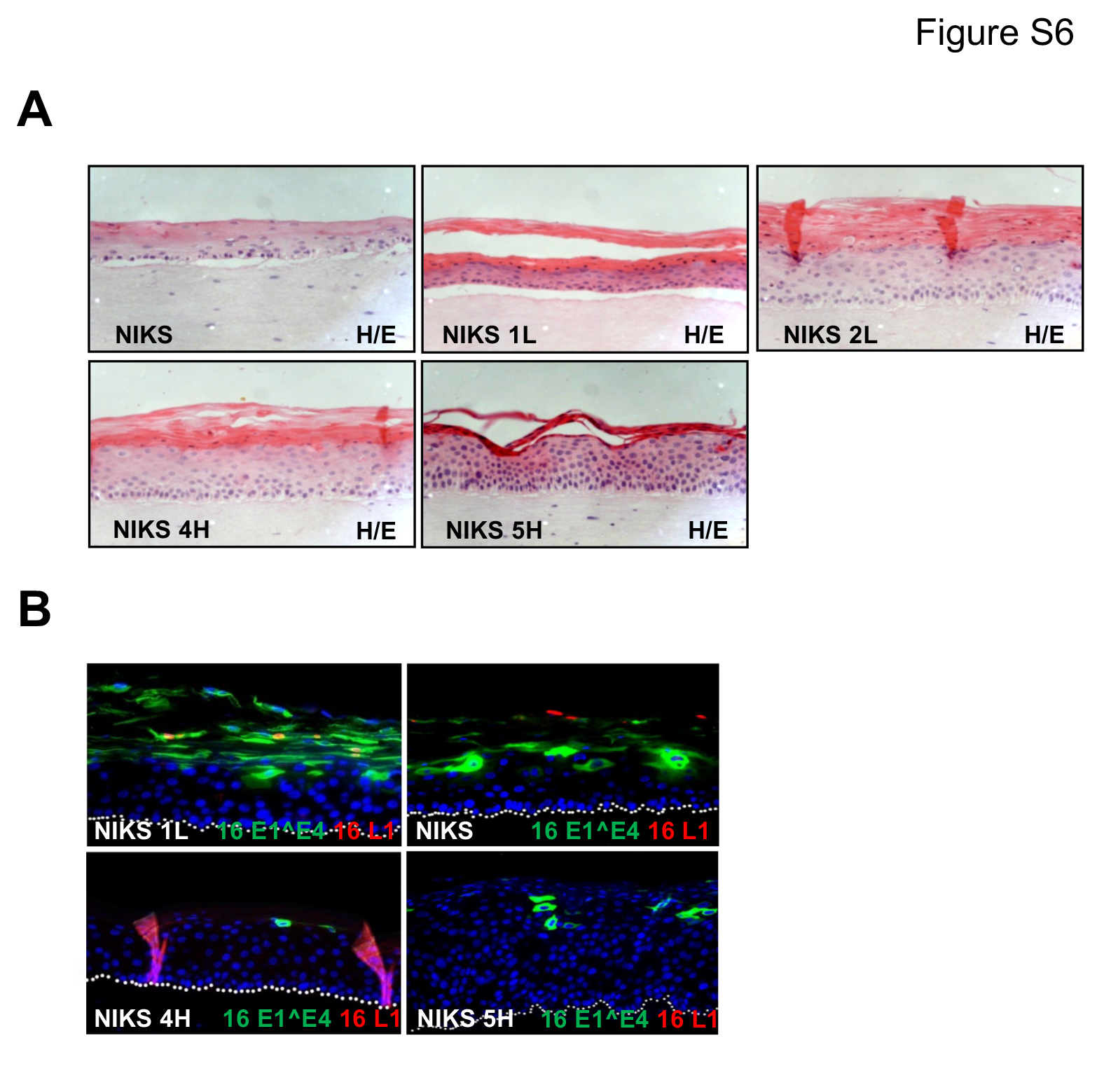

Supplement: Supplementary file 8 — Figure S6. Histological and molecular verification of episomal HPV‐16 rafts and LXSN HPV‐16 E6 and E7 rafts. (A) Haematoxylin and eosin‐stained sections of raft cultures prepared from NIKS or NIKS HPV‐16 clonal lines analysed in Figure 4. (B) Expression of the HPV‐16 life cycle‐associated proteins E1^E4 and L1 were used to evaluate the life cycle status (productive or abortive) in raft cultures prepared from HPV‐16 episomal lines. [file PATH-242-448-s012.tif]

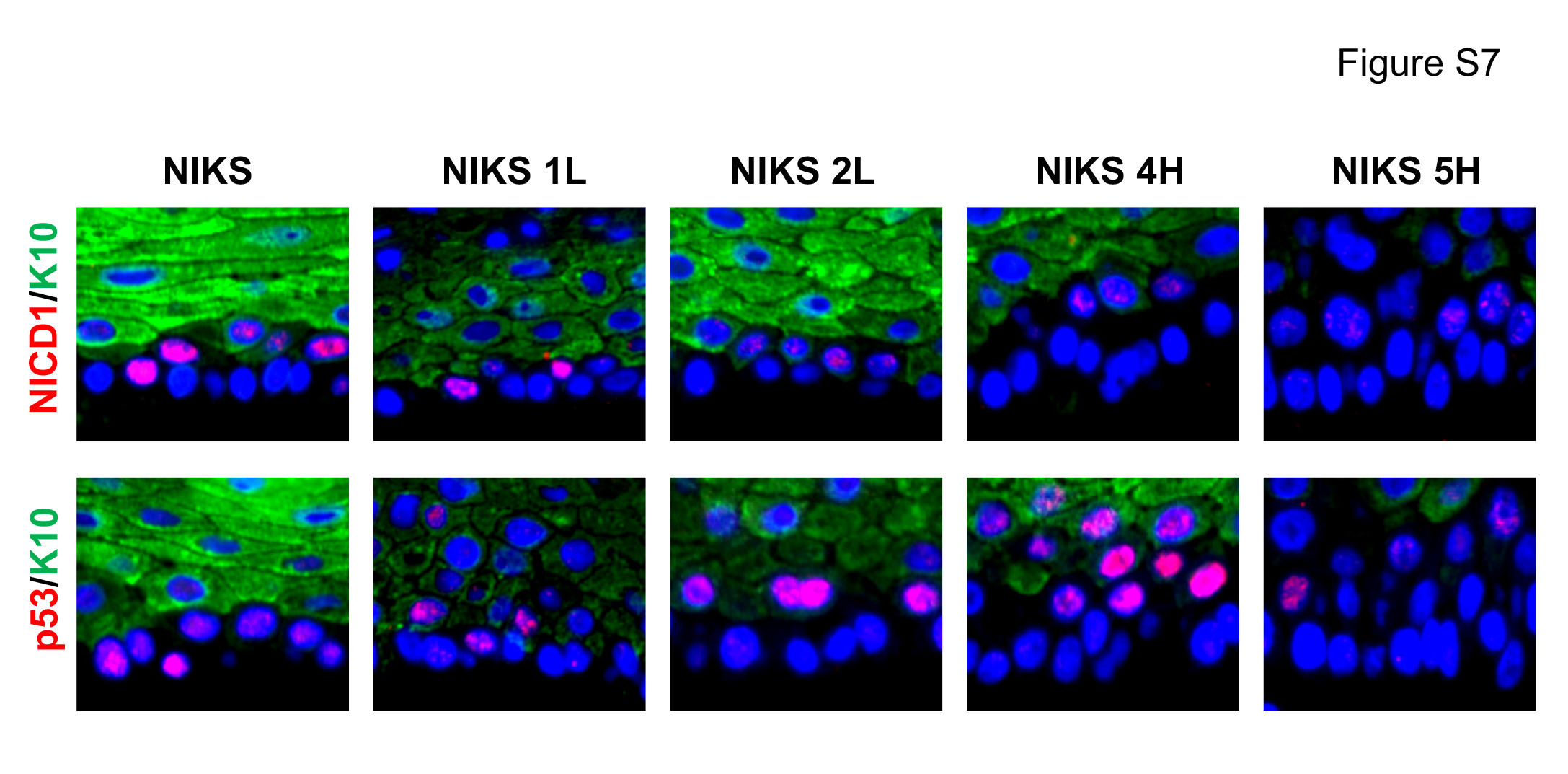

Supplement: Supplementary file 9 — Figure S7. Expression of NICD, p53, and keratin‐10 in the lower layers of NIKS, LSIL‐like, and HSIL‐like NIKS rafts. Images of individual raft cultures stained as detailed in Figure 4 were acquired at higher magnification (40×) to show differences in the appearance of p53, NICD, and keratin‐10 in the lower epithelial layers of normal and HPV‐16 NIKS raft cultures. [file PATH-242-448-s007.tif]

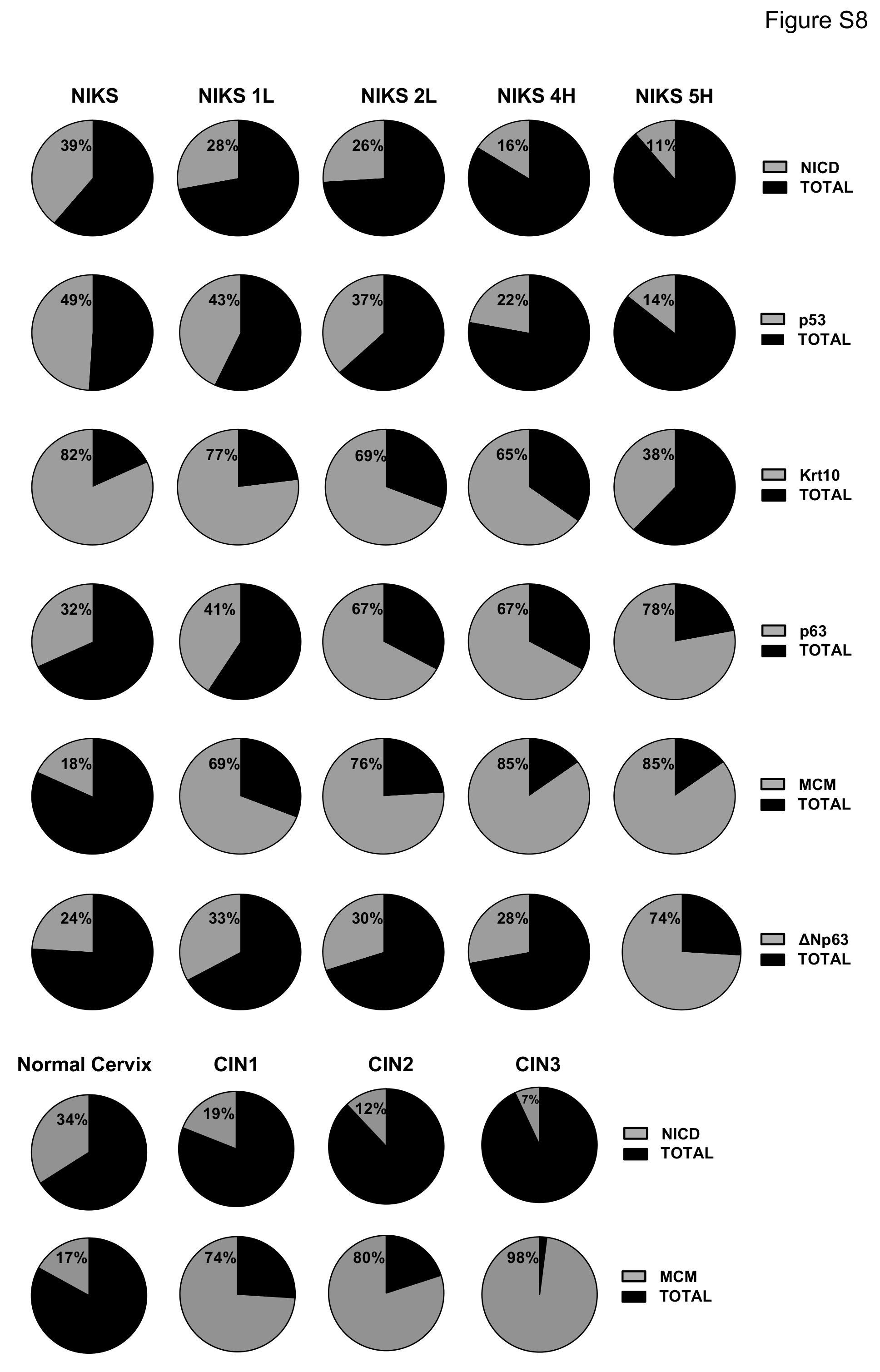

Supplement: Supplementary file 10 — Figure S8. Quantification of the protein expression patterns in the raft epithelium. For each of the indicated phenotypes, the surface area of the raft or cervical epithelium showing positive staining for each indicated protein was quantified and plotted as a percentage of the total raft area. Surface areas were extrapolated based on the number of pixels of each image. [file PATH-242-448-s009.tif]

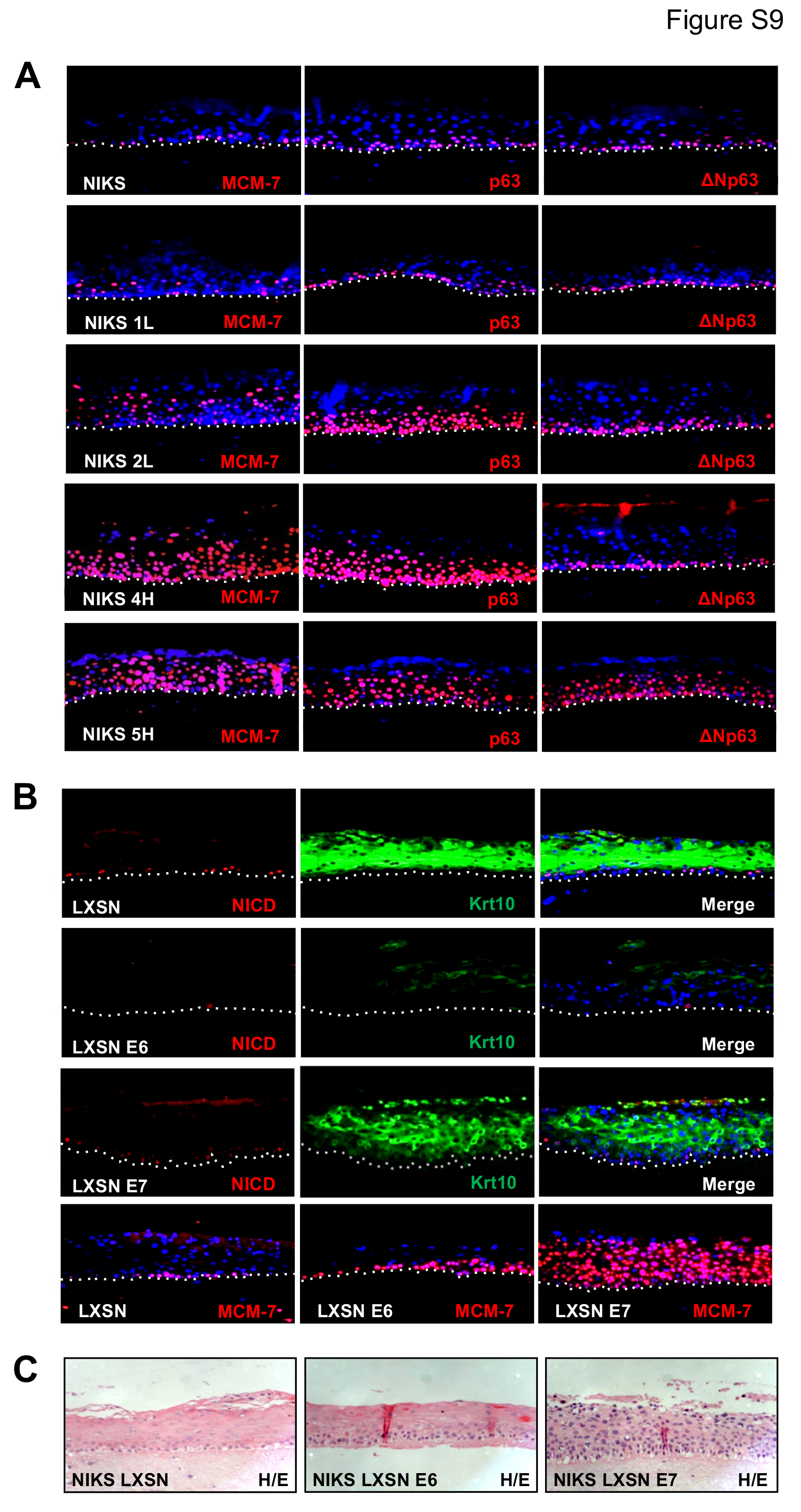

Supplement: Supplementary file 11 — Figure S9. Correlation of the differentiation status of HPV‐16 NIKS and LXSN 16E6 and 16E7 raft cultures with the expression of markers for cell cycle entry and the basal layer. (A) Representative images of the pattern of MCM7, p63, and ΔNp63 expression in raft cultures of NIKS or NIKS HPV‐16 episomal lines as in panels A–C. The fluorescence signal for MCM7, p63, and ΔNp63 was amplified with TMR. All sections were counterstained with DAPI. (B) Representative images showing the expression of NICD and Krt10 or MCM7 in raft cultures prepared from control (LXSN) NIKS or NIKS expressing either HPV‐16 E6 or E7. All sections were counterstained with DAPI. (C) Haematoxylin and eosin‐stained sections of raft cultures prepared from NIKS LXSN or NIKS expressing either HPV‐16 E6 or E7 analysed in panel B. [file PATH-242-448-s002.tif]

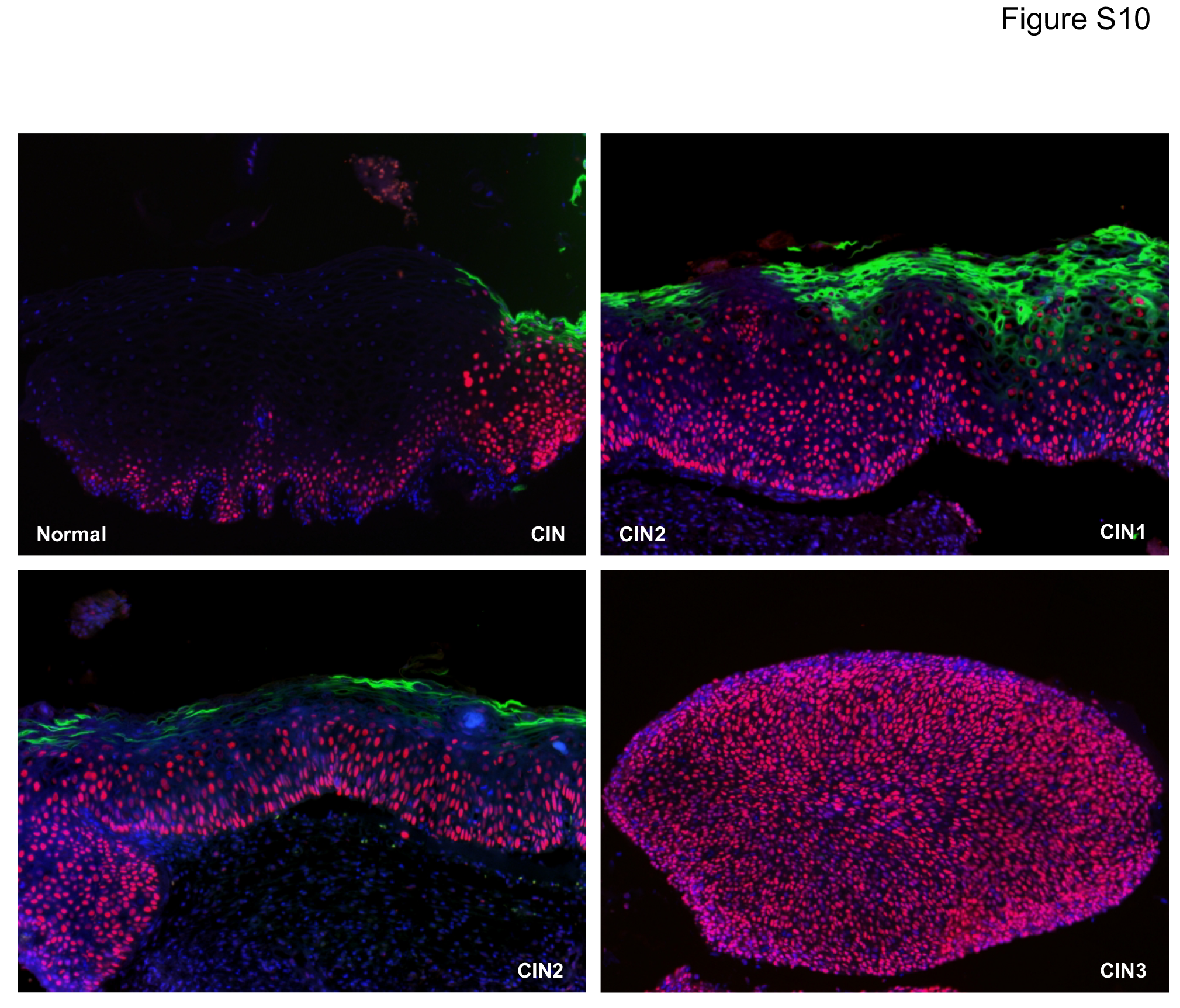

Supplement: Supplementary file 12 — Figure S10. Association of cervical phenotype with molecular markers of cell cycle entry and HPV life cycle. The CIN grading of individual cervical tissue sections described in Figure 6 was correlated with the expression of the marker for cell cycle activity MCM2 (red) and the marker of life cycle status HPV E1^E4 (green). All sections were counterstained with DAPI. [file PATH-242-448-s005.tif]
